# A network integration approach for drug-target interaction prediction and computational drug repositioning from heterogeneous information

**DOI:** 10.1038/s41467-017-00680-8

**Published:** 2017-09-18

**Authors:** Yunan Luo, Xinbin Zhao, Jingtian Zhou, Jinglin Yang, Yanqing Zhang, Wenhua Kuang, Jian Peng, Ligong Chen, Jianyang Zeng

**Affiliations:** 1 Institute for Interdisciplinary Information Sciences, Tsinghua University, Beijing, 100084 China; 2 Department of Computer Science, University of Illinois at Urbana-Champaign, Urbana, IL 61801 USA; 3 School of Pharmaceutical Sciences, Tsinghua University, Beijing, 100084 China; 4 Collaborative Innovation Center for Biotherapy, State Key Laboratory of Biotherapy and Cancer Center, West China Hospital, West China Medical School, Sichuan University, Chengdu, 610041 China

## Abstract

The emergence of large-scale genomic, chemical and pharmacological data provides new opportunities for drug discovery and repositioning. In this work, we develop a computational pipeline, called DTINet, to predict novel drug–target interactions from a constructed heterogeneous network, which integrates diverse drug-related information. DTINet focuses on learning a low-dimensional vector representation of features, which accurately explains the topological properties of individual nodes in the heterogeneous network, and then makes prediction based on these representations via a vector space projection scheme. DTINet achieves substantial performance improvement over other state-of-the-art methods for drug–target interaction prediction. Moreover, we experimentally validate the novel interactions between three drugs and the cyclooxygenase proteins predicted by DTINet, and demonstrate the new potential applications of these identified cyclooxygenase inhibitors in preventing inflammatory diseases. These results indicate that DTINet can provide a practically useful tool for integrating heterogeneous information to predict new drug–target interactions and repurpose existing drugs.

## Abstract (web summary)

Network-based data integration for drug–target prediction is a promising avenue for drug repositioning, but performance is wanting. Here, the authors introduce DTINet, whose performance is enhanced in the face of noisy, incomplete and high-dimensional biological data by learning low-dimensional vector representations.

## Introduction

Computational prediction of drug–target interactions (DTIs) has become an important step in the drug discovery or repositioning process, aiming to identify putative new drugs or novel targets for existing drugs. Compared to in vivo or biochemical experimental methods for identifying new DTIs, which can be extremely costly and time-consuming^1^, in silico or computational approaches can efficiently identify potential DTI candidates for guiding in vivo validation, and thus significantly reduce the time and cost required for drug discovery or repositioning. Traditional computational methods mainly depend on two strategies, including the molecular docking-based approaches^2, 3^ and the ligand-based approaches^4^. However, the performance of molecular docking is limited when the 3D structures of target proteins are not available, while the ligand-based approaches often lead to poor prediction results when a target has only a small number of known binding ligands.

In the past decade, much effort has been devoted to developing the machine learning-based approaches for computational DTI prediction. A key idea behind these methods is the “guilt-by-association” assumption, that is, similar drugs may share similar targets and vice versa. Based on this intuition, DTI prediction is often formulated as a binary classification task, which aims to predict whether a DTI is present or not. A straightforward classification-based approach is to consider known DTIs as labels and incorporate chemical structures of drugs and primary sequences of targets as input features (or kernels). Most existing prediction methods mainly focus on exploiting information from homogeneous networks. For example, Bleakley and Yamanishi^5^ applied a support vector machine framework to predict DTIs based on a bipartite local model (BLM). Mei et al.^6^ extended this framework by combining BLM with a neighbor-based interaction-profile inferring (NII) procedure (called BLMNII), which is able to learn the DTI features from neighbors and predict interactions for new drug or target candidates. Xia et al.^7^ proposed a semi-supervised learning method for DTI prediction, called NetLapRLS, which applies Laplacian regularized least square (RLS) and incorporates both similarity and interaction kernels into the prediction framework. van Laarhoven et al.^8, 9^ introduced a Gaussian interaction profile (GIP) kernel-based approach coupled with RLS for DTI prediction.

In addition to chemical and genomic data^10^, previous works have incorporated pharmacological or phenotypic information, such as side-effects^11, 12^, transcriptional response data^13^, drug–disease associations^14^, public gene expression data^15^ and functional data^16^ for DTI prediction. Heterogeneous data sources provide diverse information and a multi-view perspective for predicting novel DTIs. For instance, the therapeutic effects of drugs on diseases can generally reflect their binding activities to the targets (proteins) that are related to these diseases and thus can also contribute to DTI prediction. Therefore, incorporating heterogeneous data sources, e.g., drug–disease associations, can potentially boost the accuracy of DTI prediction and provide new insights into drug repositioning. Despite the current availability of heterogeneous data, most existing methods for DTI prediction are limited to only homogeneous networks or bipartite DTI models, and cannot be directly extended to take into account heterogeneous nodes or topological information and complex relations among different data sources.

Recently, several computational strategies have been introduced to integrate heterogeneous data sources to predict DTIs. A network-based approach for this purpose is to fuse heterogeneous information through a network diffusion process, and directly use the obtained diffusion distributions to derive the prediction scores of DTIs^14, 17^. A meta-path based approach has also been proposed to extract the semantic features of DTIs from heterogeneous networks^18^. A collaborative matrix factorization method has been developed to project the heterogeneous networks into a common feature space, which enables one to use the aforementioned homogeneous network-based methods to predict new DTIs from the resulting single integrated network^19^. However, these approaches generally fail to provide satisfactory integration paradigms. First, directly using the diffusion states as the features or prediction scores may easily suffer from the bias induced by the noise and high-dimensionality of biological data and thus possibly lead to inaccurate DTI predictions. In addition, the hand-engineered features, such as meta-paths, often require expert knowledge and intensive effort in feature engineering, and hence prevent the prediction methods from being scaled to large-scale data sets. Moreover, collapsing multiple individual networks into a single network may cause substantial loss of network-specific information, since edges from multiple data sources are mixed without distinction in such an integrated network.

In this paper, we present DTINet, a novel network integration pipeline for DTI prediction. DTINet not only integrates diverse information from heterogeneous data sources (e.g., drugs, proteins, diseases and side-effects) but also copes with the noisy, incomplete and high-dimensional nature of large-scale biological data by learning low-dimensional but informative vector representations of features for both drugs and proteins. The low-dimensional feature vectors learned by DTINet capture the context information of individual networks, as well as the topological properties of nodes (e.g., drugs or proteins) across multiple networks. Based on these low-dimensional feature vectors, DTINet then finds an optimal projection from drug space onto target space, which enables the prediction of new DTIs according to the geometric proximity of the mapped vectors in a unified space. We have demonstrated the integration capacity of DTINet by unifying multiple networks related to drugs and proteins, and shown that incorporating additional network information can significantly improve the prediction accuracy. In addition, through comprehensive tests, we have demonstrated that DTINet can achieve substantial performance improvement over other state-of-the-art prediction methods. Furthermore, we have experimentally validated the new interactions predicted by DTINet between three drugs and the cyclooxygenase (COX) proteins that have not been reported in the literature (to the best of our knowledge), and demonstrated the potential novel applications of these drugs in preventing inflammatory diseases. All these results demonstrate that DTINet can offer a practically useful tool to predict unknown DTIs from complex heterogeneous networks, which may provide new insights into drug discovery or repositioning and the understanding of mechanisms of drug action.

## Results

### Overview of DTINet

**Fig. 1:**
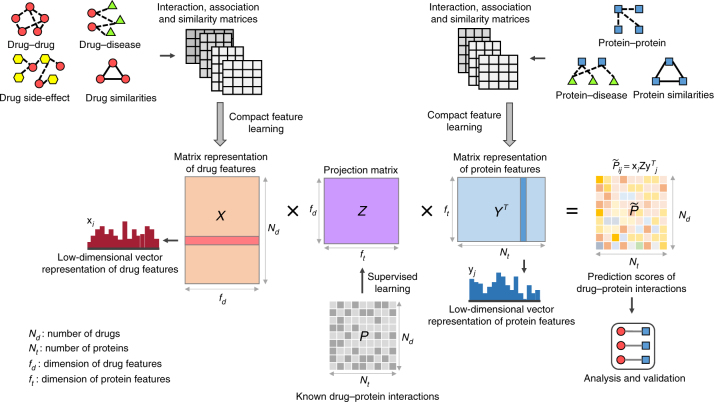
The flowchart of the DTINet pipeline. DTINet first integrates a variety of drug-related information sources to construct a heterogeneous network and applies a compact feature learning algorithm to obtain a low-dimensional vector representation of the features describing the topological properties for each node. With the learned compact features *X* and *Y* for drugs and proteins (i.e., each row in *X* and *Y* represents the feature vector of a drug and a protein, respectively), DTINet then finds the best projection from drug space onto protein space, such that the projected feature vectors of drugs are geometrically close to the feature vectors of their known interacting proteins. The projection matrix *Z* is learned to minimize the difference between the known interaction matrix *P* and *XZY* ^*T*^ (see Supplementary Note 1 for more details). After that, DTINet infers new interactions for a drug by sorting its target candidates based on their geometric proximity to the projected feature vector of this drug in the projected space. The predicted new drug–target interactions can be further analyzed and experimentally validated

**Fig. 2:**
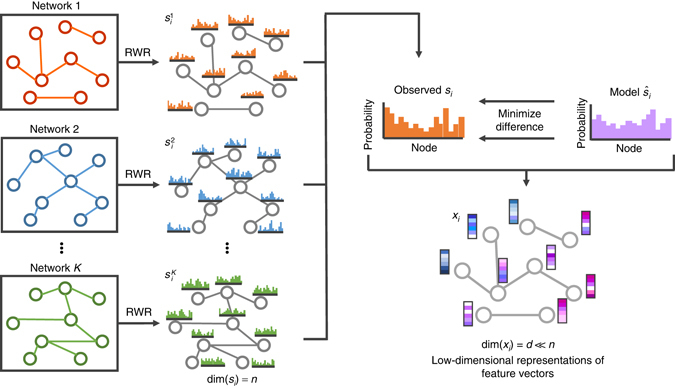
Schematic illustration of compact feature learning. The random walk with restart (RWR) algorithm is first used to compute the diffusion states of individual networks. Then the low-dimensional representations of feature vectors for individual nodes are obtained by minimizing the difference between the diffusion states *s*_*i*_ and the parameterized multinomial logistic models ${\hat s_i}$. The learned low-dimensional feature vectors encode the relational properties (e.g., similarity), association information and topological context of each node in the heterogeneous network. More details can be found in the main text

We develop a new computational pipeline, called DTINet, to predict novel DTIs and thus identify new indications of old drugs from a heterogeneous network (Supplementary Fig. 1). As an overview (Fig. 1), DTINet integrates diverse information from heterogeneous network by first combining the network diffusion algorithm (random walk with restart, RWR^20^) with a dimensionality reduction scheme (diffusion component analysis, DCA^21–23^), to obtain informative, but low-dimensional vector representations of nodes in the network (Fig. 2). Such a process is also called compact feature learning. Intuitively, the low-dimensional feature vector obtained from this process encodes the relational properties (e.g., similarity), association information and topological context of each drug (or protein) node in the heterogeneous network. Next, DTINet finds the best projection from drug space onto protein space, such that the mapped feature vectors of drugs are geometrically close to their known interacting targets. After that, DTINet infers new interactions for a drug by ranking its target candidates according to their proximity to the projected feature vector of this drug (Fig. 1). A key insight of our approach is that the drugs (or proteins) with similar topological properties in the heterogeneous network are more likely to be functionally correlated. For example, those drugs that are close in the directions of their feature vectors are more likely to act on the same targets, and vice versa. This intuition allows us to predict unknown DTIs by fully exploiting our previous knowledge about known DTIs. More details of the DTINet pipeline can be found in Methods and Supplementary Note 1.

### DTINet yields accurate DTI prediction

**Fig. 3:**
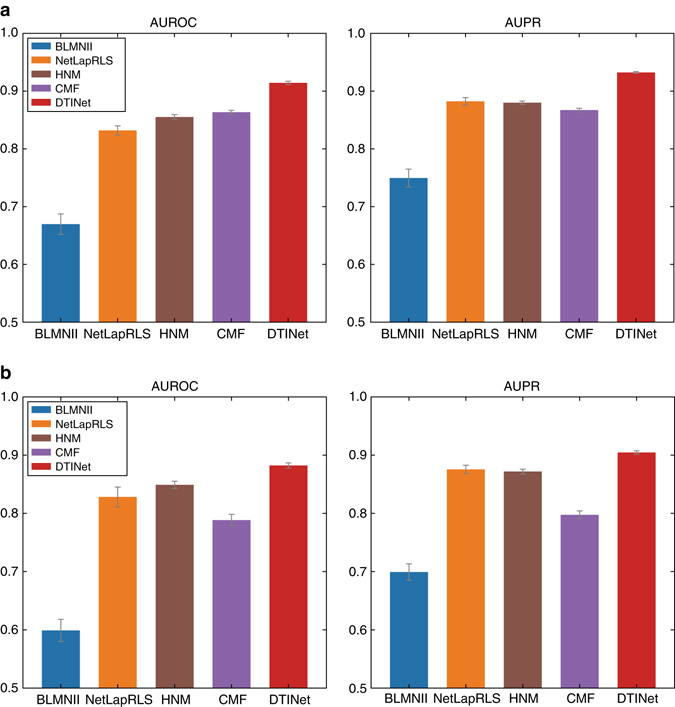
DTINet outperforms other state-of-the-art methods for DTI prediction. We performed a ten-fold cross-validation procedure to compare the prediction performance of DTINet to that of four state-of-the-art DTI prediction methods, i.e., HNM, CMF, and the extended versions of BLMNII and NetLapRLS (see Supplementary Note 1). Performance of each method was assessed by both the area under ROC curve (AUROC) and the area under precision-recall curve (AUPRC). **a** All methods were trained and tested on the original collected data set (see the main text), without removing any homologous protein. **b** All methods are trained and tested on a modified data set, in which homologous proteins were excluded. A pair of two proteins are said to be homologous if their sequence identity score is above 40%. All results were summarized over 10 trials and expressed as mean ± SD

We first evaluated the prediction performance of DTINet using a ten-fold cross-validation procedure, in which a randomly chosen subset of 10% of the known interacting drug–target pairs and a matching number of randomly sampled non-interacting pairs were held out as the test set, and the remaining 90% known interactions and a matching number of randomly sampled non-interacting pairs were used to train the model. We compared DTINet with four state-of-the-art methods for DTI prediction, including BLMNII^6^, NetLapRLS^7^, HNM^14^ and CMF^19^ (Supplementary Note 1). Our comparative results showed that DTINet consistently outperformed other existing methods, with 5.9% higher AUROC and 5.7% higher AUPR than the second best method (Fig. 3a). Compared to HNM, which predicts DTIs based on a modified version of random walk in a complete heterogeneous network, DTINet achieved 6.9% higher AUROC and 5.9% higher AUPR, presumably because HNM only uses the original diffusion states for prediction, which is not entirely accurate, while DTINet applies a novel dimensionality reduction on the diffusion states and thus is able to capture the underlying structural properties of the heterogeneous network.

To mimic a practical situation in which a DTI matrix is often sparsely labeled with only a few known DTIs, we also performed two additional cross-validation tests, in which the negative set in the test data contained either the negative samples nine times more than the positive ones or all the remaining non-interacting drug–target pairs that were not in the training data (Supplementary Fig. 2). In these two settings with imbalanced data sets, the known DTIs (i.e., positive samples) composed only 10% and 0.18% of the whole data set, respectively. In these two tests, although the AUPR scores of all methods dropped when compared to the previous test (Fig. 3a), we observed that DTINet still achieved much higher AUPR than other methods (Supplementary Fig. 2). As studied in previous works^8, 24^, AUROC is likely to be an overoptimistic metric to evaluate the performance of a prediction algorithm, especially on highly skewed data, while AUPR can provide a better assessment in this scenario. Thus, the noticeable performance improvement of DTINet in terms of AUPR over other prediction methods demonstrated its superior ability in predicting new DTIs in the sparsely labeled networks.

The originally collected data sets (Methods) may contain homologous proteins or similar drugs, which raised a potential concern that the good performance of prediction methods might result from easy predictions. To investigate this issue, we performed the following additional tests (Fig. 3b and Supplementary Fig. 3a–d): (1) the removal of the DTIs involving homologous proteins (sequence identity scores >40%); (2) the removal of the DTIs with similar drugs (Tanimoto coefficients >60%); (3) the removal of the DTIs with the drugs sharing similar side-effects (Jaccard similarity scores >60%); (4) the removal of the DTIs with the drugs or proteins associated with similar diseases (Jaccard similarity scores >60%); and (5) the removal of the DTIs with either similar drugs (Tanimoto coefficients >60%) or homologous proteins (sequence identity scores >40%). In the above tests, the removal operations can further reduce the potential redundancy in the DTIs that may cause the inflated evaluation performance in cross-validation. The test results under the above settings showed that DTINet was robust against the removal of homologous proteins or similar drugs in training data and still consistently outperformed other methods (Fig. 3b and Supplementary Fig. 3). We also removed the DTIs with homologous proteins in a skewed data set in which the known DTIs composed only 10% of the whole data set, and observed similar results (Supplementary Fig. 3e). Other threshold values for drug similarity scores and protein identity scores were also evaluated, and similar trends were observed (results not shown). Taken together, these results demonstrated that DTINet can still achieve decent performance and outperform other prediction methods even without the presence of similar drugs or targets.

The random split of training and test data in the conventional cross-validation procedure may raise another concern due to “popular” drugs^25^. Since the drugs that are well-connected (i.e., with large degrees) to proteins in the DTI network tend to be predicted more easily and thus may result in the inflated high recall rates, it is important to seek a proper evaluation procedure and metric to assess the performance of prediction methods under more realistic drug repositioning scenarios. To this end, we first hid all the DTIs in which the related drugs have new mechanism of actions discovered within the 5 years as of the time that the DrugBank database Version 3.0 (which was used to construct our heterogeneous network) was released. According to this criterion, we held out 255 DTIs related to 79 drugs as the test set. As in the previous works^25, 26^, we used “recall @ top-*k*” as the evaluation metric, which is defined as the fraction of true interacting targets that were retrieved in the list of top-*k* predictions for a drug. The motivation of using this metric was that a method that can accurately recover the true interacting targets in the list of top-*k* predictions is generally desired and useful for the downstream experimental validation. We found that DTINet achieved much better performance than other methods in recovering more true interacting targets for a given drug at different values of rank *k* (Supplementary Fig. 4a). In the second setting, we evaluated the prediction performance of different methods on those singleton drugs, which have only one interacting known target in our data set. Such a setting can be considered as a difficult case in computational drug repositioning, in that all these singleton drugs have no known interacting targets available in the training data. This setting can be used to assess the performance of prediction methods on those DTIs that are relatively less well studied and characterized. We observed that DTINet retrieved ~50% of true DTIs for a singleton drug in the list of top 150 predictions, in contrast to a fraction less than 28% for other methods (Supplementary Fig. 4b). Overall, the test results under the above two settings demonstrated the superior ability of DTINet in integrating heterogeneous information into the prediction of new DTIs in real drug repositioning scenarios and serving as a practically useful tool for computational drug repositioning and drug discovery.

DTINet copes with the noise and incompleteness in the high-dimensional data by learning the compact representations that capture the most explanatory features. To directly evaluate the robustness of DTINet under this setting, we randomly perturbed the topological structures in the network data. In particular, 10% randomly sampled edges in the heterogeneous network were perturbed, by adding new edges or deleting existing interaction (or association) edges. Compared to NetLapRLS, DTINet achieved more robust performance against the incompleteness or noise in the network data (Supplementary Fig. 5). This result demonstrated the robustness of DTINet in extracting the relevant latent topological patterns even under the setting of noisy network data.

Our further comparative study showed that integrating multiple networks derived from the feature vectors of drugs or proteins by DTINet can greatly improve the prediction performance over individual single networks (Supplementary Fig. 6). Our comparison demonstrated that, even without multiple networks integration, DITNet still outperformed the state-of-the-art single network-based method NetLapRLS on individual similarity networks. This result emphasized DTINet’s ability to fully exploit useful topological information from high-dimensional and noisy network data via a compact learning procedure, even only given a single network as input. In addition, we observed that DTINet achieved much better prediction performance than the extended version of NetLapRLS, when integrating multiple networks into a heterogeneous one. These results indicated that integrating multiple networks into DTI prediction is not a trivial task, while the network integration procedure of DTINet can simultaneously and effectively capture the underlying topological structures of multiple networks, leading to the improved accuracy of DTI prediction. Moreover, in terms of time complexity, DTINet runs fast and only takes roughly cubic time (see Supplementary Note 1).

### DTINet identifies novel DTIs

**Fig. 4:**
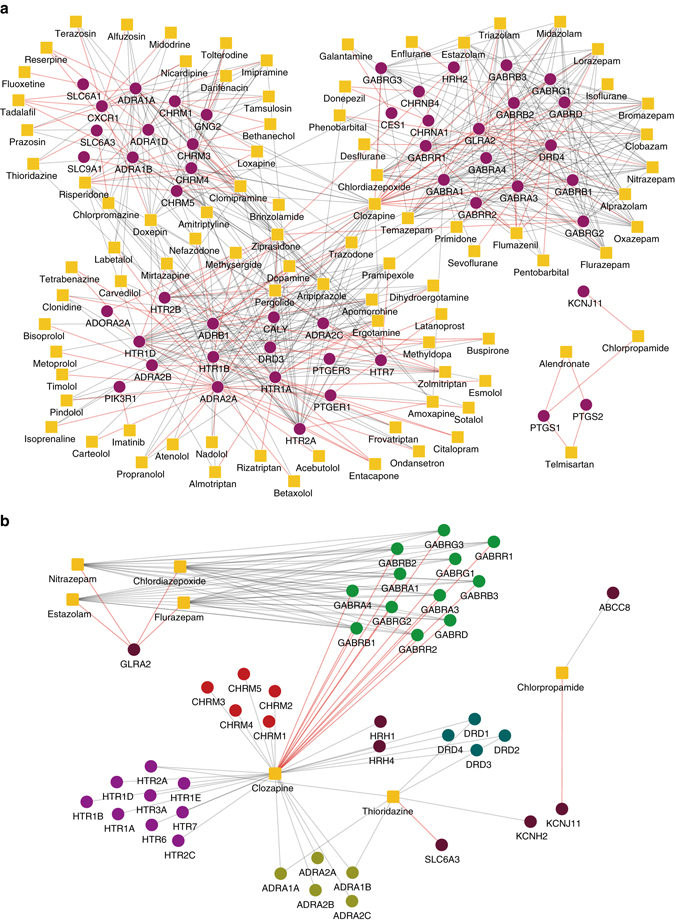
Network visualization of the drug–target interactions predicted by DTINet. **a** Visualization of the overall drug–target interaction network involving the top 150 predictions (also see Supplementary Data 1). Target and drugs are shown in *purple circles* and *yellow boxes*, respectively. **b** Network visualization of several examples of novel DTI predictions which can be supported by known experimental or clinical evidence in the literature. The drugs are shown in *yellow boxes*, white different families of their interacting targets are shown in *circles* with different colors. In both **a**, **b**, known drug–target interactions are marked by *grey edges*, while the new predicted interactions are shown by *red edges*

We also predicted the novel DTIs using the whole heterogeneous network (in which drug and targets have at least one known interacting pair) as training data and outputted the list of top predictions (Supplementary Datas 1, 2). We excluded those easy predictions in which the targets have the sequence identity scores above 40% from the homologous proteins in training data. Among the list of top 150 predictions (Fig. 4a and Supplementary Data 1), we found that many of them can also be supported by the previously known experimental or clinical evidence in the literature (Fig. 4b and Supplementary Data 3). For example, new predictions showed that clozapine can act on the gamma-aminobutyric acid (GABA) receptors, an essential family of channel proteins that modulate the cognitive functions (Fig. 4b). This new prediction can be supported by the previous studies which showed that clozapine can have a direct interaction with the GABA B-subtype (GABA-B) receptors^27^ and antagonize the GABA A-subtype (GABA-A) receptors in the cortex^28^. More examples of such novel predictions that can be supported from the previous studies in the literature can be found in Supplementary Data 3.

Next, we focused on those novel DTIs among the list of top 150 predictions from DTINet, for which we rarely found known experimental support in the literature. Among the list of these top 150 predictions, most of the new predicted DTIs were relevant (i.e., connected) to the previously known interactions except the interactions between three drugs, including telmisartan, chlorpropamide and alendronate, and the prostaglandin-endoperoxide synthase proteins, which are also called COX proteins (Fig. 4a). COX is a family of enzymes responsible for prostaglandin biosynthesis^29^, and mainly includes COX-1 and COX-2 in human, both of which can be inhibited by nonsteroidal anti-inflammatory drugs (NSAIDs)^30^. Apparently, it was difficult to use the correlations between nodes within the DTI network to explain the predicted interactions between these three drugs and the COX proteins. On the other hand, these new DTIs had relatively high prediction scores in the list of the top 150 predictions (Supplementary Data 1). In addition, the COX proteins provide a class of important targets in a wide range of inflammatory diseases^31^. Despite the existence of numerous known NSAIDs used as COX inhibitors, many of them are associated with the cardiovascular side-effects^32, 33^. Thus, it is always important to identify alternative COX inhibitors from existing drugs with fewer side-effects. Given these facts, it would be interesting to see whether the predicted interactions between these three drugs and the COX proteins can be further validated.

Among the aforementioned three drugs, telmisartan has been known as an angiotensin II receptor antagonist that can be used to treat hypertension^34^, chlorpropamide has been known as a sulfonylurea drug that acts by increasing insulin to treat type 2 diabetes mellitus^35^, and alendronate has been known as a bisphosphonate drug mainly used for treating bone disease, such as osteoporosis and osteogenesis imperfect^36, 37^. Despite our current understanding about the functions of COX-1 and COX-2 proteins and the known indications of telmisartan, chlorpropamide and alendronate, it still remains largely unknown whether these three drugs can also interact with the COX proteins. According to the top 150 predictions by DTINet (Fig. 4a and Supplementary Data 1), these three drugs can act on the COX proteins. We will further present our validation results on the predicted interactions between these three drugs and COX proteins in the next sections.

### Computational docking suggests binding modes

**Fig. 5:**
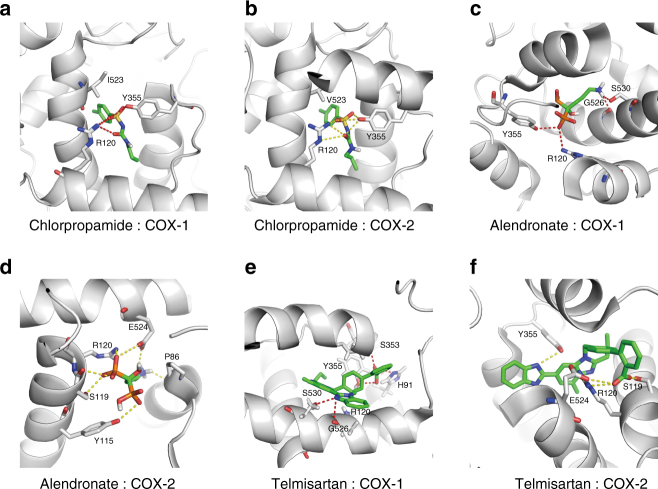
The docked poses for the predicted interactions between three drugs (i.e., chlorpropamide, alendronate and telmisartan) and the COX proteins (i.e., COX-1 and COX-2). **a** Chlorpropamide vs. COX-1; **b** Chlorpropamide vs. COX-2; **c** Alendronate vs. COX-1; **d** Alendronate vs. COX-2; **e** Telmisartan vs. COX-1; **f** Telmisartan vs. COX-2. The protein structures of COX-1 and COX-2 were downloaded from the Protein Data Bank (PDB IDs 3kk6 and 3qmo for COX-1 and COX-2, respectively). The structures of the small molecules were obtained from the ZINC^61^. The docking program Autodock^3^ was used for the docking modeling. Hydrogen bonds were computed by PyMOL^70^ and represented by the *red* and *yellow dashed lines* in COX-1 and COX-2, respectively

Our docking studies (Methods) showed that the three drugs (i.e., telmisartan, alendronate and chlorpropamide) were able to dock to the structures of both COX-1 (PDB ID: 3kk6) and COX-2 (PDB ID: 3qmo), and displayed different binding patterns (Fig. 5). In particular, all three drugs were fitted into the active sites of both COX-1 and COX-2. More specifically, chlorpropamide displayed similar configurations when binding to COX-1 and COX-2 (Fig. 5a, b), by forming hydrogen bonds with both residues R120 and Y355, which created a conserved pocket as in those for common NSAIDs^38, 39^. On the other hand, the substitution of V119 in COX-1 by S119 in COX-2 allowed the formation of a different hydrogen bond network in the binding pocket. Moreover, telmisartan and alendronate interacted with residue S530 in addition to residues R120 and Y355 when docked to COX-1 (Fig. 5c, e), while they were both able to bind to residue S119 when docked to COX-2 (Fig. 5d, f). Thus, a subtle difference between the binding pockets of those two enzymes may result in different binding modes even for the same drug. These docking results may provide important hints for understanding the structural basis of the predicted DTIs and thus help reveal the underlying molecular mechanisms of drug action.

### Experimental validation of the top-ranked predictions

**Fig. 6:**
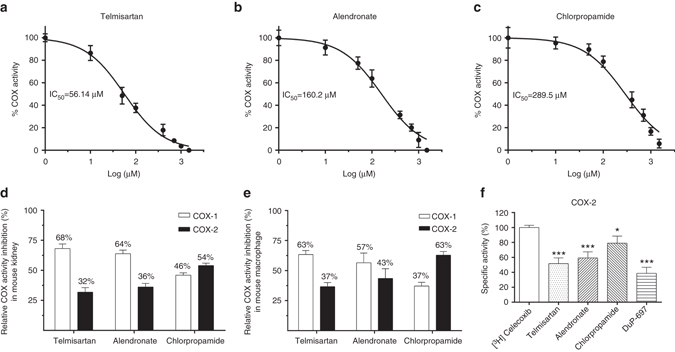
Inhibitory effects of telmisartan, alendronate and chlorpropamide on COX activity measured by COX inhibition assays. **a**–**c** The inhibition rates of telmisartan, alendronate and chlorpropamide measured by the COX fluorescent activity assays on the mouse kidney lysates. **d**, **e** The relative COX activity inhibition rates of telmisartan, chlorpropamide and alendronate on COX-1 and COX-2, measured by the COX fluorescent activity assays on the tissue extracts from both kidney **d** and macrophage **e** lysates. **f** The results on the competitive binding of [^3^H] celecoxib for different COX-2 inhibitors measured by the radioligand-based binding assays. Control: the radioactivity of the sample with [^3^H] celecoxib only. *: *P* < 0.05, **: *P* < 0.01, ***: *P* < 0.001, Newman–Keuls multiple comparison test. Here, data show the mean with the standard deviation of three independent experiments, each of which was performed with triplicates

We further sought to experimentally validate the bioactivities of the COX inhibitors predicted by DTINet (Methods). First, we tested their inhibitory potencies on the mouse kidney lysates using the COX fluorescent activity assays. Similar dose-dependent repression of COX activity was observed for the three drugs (Fig. 6a–c). The IC_50_ values of telmisartan, alendronate and chlorpropamide for COX activity were measured at 56.14, 160.2 and 289.5 μM, respectively. The measured IC_50_ values of the three drugs especially telmisartan were comparable to those of many common NSAIDs, such as celecoxib (COX-1: 82 μM; COX-2: 6.8 μM), ibuprofen (COX-1: 12 μM, COX-2: 80 μM) and rofecoxib (COX-1: >100 μM; COX-2: 25 μM)^40, 41^. Probably alendronate and chlorpropamide were relatively weak inhibitors of COX. It is worth noting that the order of the experimentally measured IC_50_ values of these three drugs was consistent with the ranking of prediction scores in DTINet (Supplementary Data 1).

Next, the tissue extracts from the mouse kidney and the peritoneal macrophages were used for COX selective inhibition assays. Relative inhibition of COX-1 and COX-2 activities were distinguished using SC-560, a potent and selective COX-1 inhibitor, and Dup-697, a potent and time-dependent of COX-2 inhibitor, respectively. Overall, the assays on the tissue extracts from mouse kidney showed that telmisartan and alendronate had slightly higher inhibition rates on COX-1 (68 and 64%, respectively) than COX-2 (32 and 36%, respectively), while chlorpropamide had a slightly higher inhibition rate on COX-2 (54%) than on COX-1 (46%) (Fig. 6d). Similar patterns of COX inhibition selectivity with these drugs were also observed in the peritoneal macrophages (Fig. 6e). To further evaluate the selectivity of these predicted drugs on COX-1 and COX-2, we also used the human recombinant enzyme assays to measure the levels of PGE 2 under COX-1 and COX-2 catalyzes, respectively. The assay results showed that telmisartan, alendronate and chlorpropamide had IC_50_ values of 41.97, 90.73 and 223.5 μM for COX-1, respectively, and 91.75, 184.1 and 151.9 μM for COX-2, respectively (Supplementary Fig. 7). Such results were also consistent with the IC_50_ values measured by the previous selective inhibition assays (Fig. 6d, e). These validation results were also in line with the observation that the predicted scores of these novel DTIs output by DTINet were actually not that far away (Supplementary Data 1). Overall, the above inhibition assays showed that these three drugs identified by DTINet had a certain level of inhibition affinity and may act as non-selective COX inhibitors on the family of COX proteins.

To further validate the predicted DTIs, we also applied the radioactive isotope labeled with the COX-2 selective inhibitor [^3^H] celecoxib for the competitive binding assays. As shown in Fig. 6f, telmisartan, alendronate, chlorpropamide and DuP-697 (a standard COX-2 inhibitor) inhibited the binding of [^3^H] celecoxib to COX-2 by about 48.33, 40.67, 14.00 and 61.33% at their IC_50_ concentrations, respectively. These assay results provided another piece of evidence to confirm that these drugs may have direct interactions with the COX proteins.

The COX inhibitors have been extensively used as NSAIDs, thus we further tested the effects of the above three drugs on inflammatory responses and thus examined their potential applications in treating inflammatory diseases. Lipopolysaccharide (LPS) was used to stimulate the cultured peritoneal macrophages for the cellular inflammation model. In addition to those three drugs (i.e., telmisartan, chlorpropamide and alendronate) predicted by DTINet, we also considered the potent COX-2 inhibitor Dup-697 and the well-known NSAID ibuprofen for comparison.

**Fig. 7:**
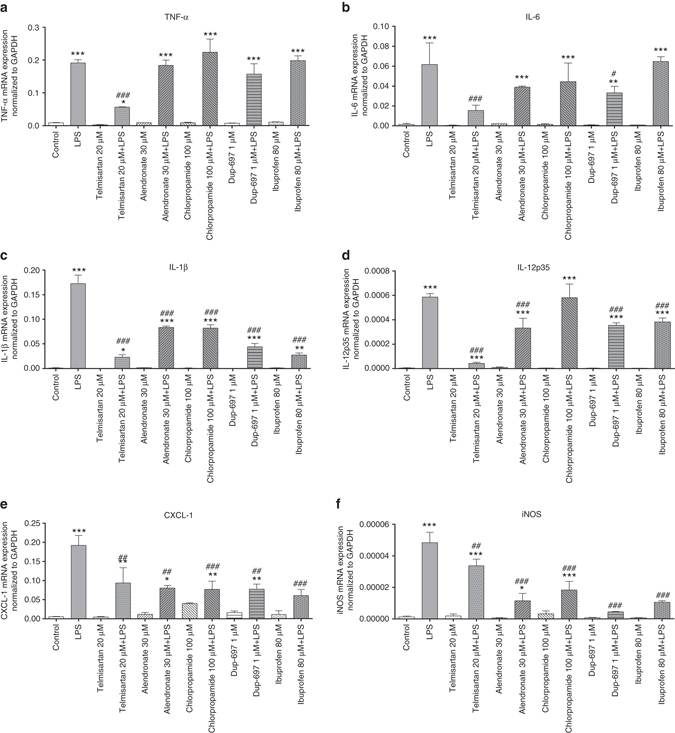
The real-time PCR (RT-PCR) analyses of the proinflammatory factors on the LPS-stimulated macrophages. **a**–**f** The RT-PCR analysis of mRNA expressions of TNFα, IL-6, IL-1β, IL-12p35, CXCL-1 and iNOS normalized relative to that of GAPDH, respectively. Control, macrophages without LPS treatment. *: *P* < 0.05; **: *P* < 0.01; ***:*P* < 0.001, compared to the samples without LPS treatment. ##: *P* < 0.01; ###:*P* < 0.001, compared to the samples treated with LPS. *n* = 3. Newman–Keuls multiple comparison test was used. Here, data show the mean with the standard deviation of three independent experiments, each of which was performed with triplicates. The concentrations of the COX inhibitors were determined according to the indications of the assay kits and the previous binding studies in the literature (see Methods)

A large amount of proinflammatory factors can be generated during the inflammation process^42^. We consequently tested whether the three drugs can suppress the expression of various inflammatory factors in response to LPS stimulation (Fig. 7). For tumor necrosis factor-α (TNF-α) and interleukin (IL)-6, telmisartan exhibited strong inhibitory effect on the LPS induced expression (Fig. 7a, b). Meanwhile, the induction of the important cytokine IL-1β was also attenuated by each of the three drugs in the peritoneal macrophages (Fig. 7c). In particular, telmisartan displayed the strongest suppression effect on IL-1β among all COX inhibitors. For IL-12p35, although both alendronate and telmisartan significantly inhibited its production induced by LPS, telmisartan had much stronger suppression effect than other COX inhibitors (Fig. 7d). The LPS-induced production of the immunological defensive factors such as CXCL-1 and inducible nitric oxide synthase were significantly restrained by the treatment of any of these three drugs (Fig. 7e, f), which was similar to the results of both Dup-697 and ibuprofen. In summary, these results showed that telmisartan, chlorpropamide and alendronate can reduce the expressions of proinflammatory factors in mouse peritoneal macrophages. The observed anti-inflammation effects of these three drugs further extended the above inhibition assay studies and demonstrated their potential applications in preventing inflammatory disease.

Taken together, the above experimental assays validated the novel interactions between the three drugs (i.e., telmisartan, alendronate and chlorpropamide) and the COX proteins predicted by DTINet, which further demonstrated the accuracy of its prediction results and thus provided strong evidence to support its excellent predictive power. In addition, the experimentally validated interactions between these three drugs and the COX proteins can provide great opportunities for drug repositioning, i.e., finding the new functions (i.e., anti-inflammatory effects) of these drugs, and offer new insights into the understanding of their molecular mechanisms of drug action or side-effects of these drugs.

## Discussion

Recent advances in large-scale experimental approaches, e.g., mass spectrum-based methods^43–46^, have made great contribution to drug development and drug target identification with high throughput and accuracy. Nevertheless, these methods can only test one chemical at a time to determine the interacting proteins. In addition, they are still costly even in a high-throughput manner. Comparing to these proteomics-based methods, computational approaches can allow high-throughput prediction for both drugs and targets, learning their intrinsic features and inferring the interactions between all potential drug–target pairs simultaneously. Based on the computational methods, we can identify a list of promising candidates and thus greatly reduce the huge search space of drug–target pairs that need to be validated by wet lab experiments.

Recently, Guney et al.^47^ showed that the network-based proximity of known drug targets and disease-associated proteins on the interactome can provide a good indicator for studying drug–disease associations and drug efficacy. Cheng et al.^48^ also developed a network-based pipeline to predict new indications of existing drugs, which basically assumed that a drug can be applied to specific cancer types if the significantly mutated genes are enriched in those differentially expressed genes induced by the drug. These two methods were mainly used to study the drug–disease relationships, but did not directly provide the information of new DTIs, which instead was the major goal of our framework. Although the drug–disease relationships may provide more direct indications of existing drugs, knowing the explicit DTIs can shed light on the underlying pharmacological mechanisms, which are important for understanding both therapeutic and adverse effects of the corresponding drugs. In addition, the aforementioned two approaches simply focused on the distances between the disease-related and drug-related proteins, which would be sensitive to the incompleteness of known targets, disease genes and underlying protein–protein interactions. On the other hand, based on the systematic integration of heterogeneous network information, in principle our approach can achieve better and more robust prediction performance (e.g., with less false-positive predictions) by considering diverse information from various types of network features.

Among the three drugs whose interactions with the COX proteins have been validated experimentally in our study, telmisartan displays unique pleiotropic roles in addition to the renin–angiotensin system -inhibition effects as an angiotensin II AT1 receptor antagonist/blocker. It has been reported that telmisartan acts as a selective modulator of the peroxisome proliferator-activated receptors (PPAR-γ and -δ)^49^. Our findings probably add novel insights into its anti-inflammatory effects as a COX inhibitor. Several studies have indicated that telmisartan ameliorates the neuronal, airways and coronary plaque inflammatory responses^50, 51^. Our findings provide direct evidence to support its interaction on the COX proteins. Its inhibitory effects on COX and inflammatory cytokine production may partially explain its anti-inflammatory indications.

For chlorpropamide, a sulfonylurea to increase the secretion of insulin to treat type 2 diabetes, there are few reports about its anti-inflammatory effects. Our findings indicate that chlorpropamide can also be a COX inhibitor though with weak binding affinity (IC_50_ around 300 μM), which may have implication on its adverse drug reactions on hematological changes, such as thrombocytopenia and granulocytopenia as in the hematologic syndromes induced by other cox inhibitors^52, 53^. Alendronate, another drug that we have tested, is a bisphosphonate drug and potent inhibitor of bone resorption used for the treatment of metabolic bone diseases. Recent studies have shown that alendronate can also suppress the production of inflammatory cytokines and matrix metalloproteinases in alveolar macrophages for its anti-inflammatory effects^54^. Our findings about its COX inhibition suggest that it may interact with COX for its immunological effects. Overall, we have combined the computational analysis with experimental validation to discover novel DTIs. Our findings are particularly helpful for understanding the unknown pharmacological effects of existing drugs and identifying their potential new applications.

A future direction of our work is to include more heterogeneous network data in our framework. While we used only four domains (i.e., drugs, proteins, diseases and side-effects) of information in this work, we highlight that DTINet is a scalable framework in that more additional networks can be easily incorporated into the current prediction pipeline. Other biological entities of different types, such as gene expression, pathways, symptoms and Gene Ontology (GO) annotations, can also be integrated into the heterogeneous network for DTI prediction. Although it was only applied to predict missing DTIs in this work, DTINet is a versatile approach and definitely can also be applied to various link prediction problems, e.g., predictions of drug–side-effect associations, drug–drug interactions and protein–disease associations.

## Methods

### The DTINet pipeline

The heterogeneous network input to DTINet is constructed based on the following known information: drug–protein interactions, drug–drug interactions, drug–disease associations, drug–side-effect associations, drug–drug similarities, protein–disease associations, protein–protein interactions, and protein–protein similarities (see “Data sets” and “Construction of the heterogeneous network”). DTINet (Fig. 1) first performs a network diffusion algorithm (e.g., random walk with restart, RWR^20^) on each network to obtain a distribution (also called “diffusion state”) of each drug or protein node, which captures its topological relations to all other nodes in the heterogeneous network (Supplementary Note 1). Taking both local and global connectivity patterns into account, this step characterizes the underlying topological context and inherent connection profiles of each drug or protein node in the network. When the diffusion states of two nodes are close to each other, it implies that they are in similar positions with respect to other nodes in the network.

A key observation in the above network diffusion algorithm is that the originally computed diffusion states are not entirely accurate, in part due to the noisy, incomplete and high-dimensional nature of biological data. To cope with this issue, DTINet further applies the DCA method^21–23^ to approximate the obtained diffusion distribution by constructing a model parameterized by a low-dimensional vector representation for each drug or protein node (Fig. 1). These low-dimensional vector representations are obtained by minimizing the difference between the diffusion distributions of individual networks and the corresponding model distributions simultaneously (Fig. 2 and Supplementary Note 1). Such a process is also called compact feature learning^55^ and the resulting low-dimensional vector is also called the feature vector. Intuitively, the low-dimensional feature vector obtained from compact feature learning encodes the relational properties (e.g., similarity), association information and topological context of each drug (or protein) in the heterogeneous network. Akin to principal component analysis, which seeks the intrinsic low-dimensional linear structure of the data to best explain the variance, DCA learns a low-dimensional vector representation for all nodes such that their connectivity patterns in the heterogeneous network are best interpreted.

After obtaining the low-dimensional feature vectors of both drugs and proteins, DTINet computes the best projection from drug space onto protein space via a matrix completion method, such that the projected low-dimensional feature vectors of drugs are geometrically as close to the corresponding feature vectors of their known interacting targets as possible (Fig. 1). After that, the new interacting targets of a drug are derived based on their geometric closeness to the projected feature vector of this drug. More details about this operation can be found in Supplementary Note 1.

### Data sets

A total of four types of nodes and six types of edges, representing diverse drug-related information, were collected from the public databases and used to construct the heterogeneous network for our DTI prediction task.

We extracted the drug nodes from the DrugBank database (Version 3.0)^56^ and the protein nodes from the HPRD database (Release 9)^57^. The disease nodes were obtained from the Comparative Toxicogenomics Database^58^. The side-effect nodes were collected from the SIDER database (Version 2)^59^. In addition, we excluded those isolated nodes; in other words, we only considered those nodes which had at least one edge (see below) in the network.

We imported the known DTIs as well as drug–drug interactions from DrugBank (Version 3.0)^56^. The protein–protein interactions were downloaded from the HPRD database (Release 9)^57^. The drug–disease and protein–disease associations were extracted from the Comparative Toxicogenomics Database^58^. We also included the drug–side-effect associations from the SIDER database (Version 2)^59^.

### Construction of the heterogeneous network

Compiling various curated public drug-related databases, we constructed a heterogeneous network, which includes 12,015 nodes and 1,895,445 edges in total, for predicting missing DTIs (Supplementary Fig. 1 and Supplementary Tables 1, 2). The heterogeneous network integrates four types of nodes (i.e., drugs, proteins, diseases and side-effects) and six types of edges (i.e., drug–protein interactions, drug–drug interactions, drug–disease associations, drug–side-effect associations, protein–disease associations and protein–protein interactions). Based on chemical structures of drugs and primary sequences of proteins, we also built up multiple similarity networks (Supplementary Note 1) to further augment the network heterogeneity, providing our drug–target prediction task with diverse information and from a multiple-views perspective.

### Computational docking analyses

In our structure-based modeling studies, we used the docking program Autodock^3^ to infer the possible binding modes of the new predicted interactions between three drugs (i.e., telmisartan, chlorpropamide and alendronate) and the COX proteins. The protein structures used in our docking studies were downloaded from the Protein Data Bank^60^ (PDB IDs 3kk6 and 3qmo for COX-1 and COX-2, respectively). The three-dimensional structures of the above three drugs were obtained from the ZINC database^61^.

### Reagents

LPS (L-2360) and 4% sterile thioglycollate were purchased from Sigma-Aldrich (St Louis, MO, USA). Interferon-γ (IFN-γ) (315-05) was purchased from PeproTech (New York, NY, USA). Chlorpropamide (S4166), telmisartan (S1738), alendronate (S1624) and ibuprofen (S1638) were purchased from Selleck Chemicals (Houston, TX, USA). COX Fluorescent Activity Assay Kit (700,200), arachidonic acid (90,010), indomethacin (70,270), human recombinant COX-1 and COX-2 enzymes (17,616 and 60,122) and prostaglandin E2 (PGE 2) ELISA kit (514,010) were purchased from Cayman Chemical Company (Ann Arbor, MI, USA). COX 2 (ab62331) antibody was obtained from Abcam (Cambridge, MA, USA). [^3^H] celecoxib was purchased from Hartmann Analytics (Braunschweig, Germany).

### Animals

C57BL/6J mice (10 weeks old) were obtained from Vital River (Beijing, China) and were housed under controlled temperature (22 °C ± 2 °C) and humidity (40–60%) with a 12 h light/dark cycle. For each experiment, three mice were randomly injected intraperitoneally with 1 ml of 4% sterile thioglycollate and sacrificed 3 days later. All animal surgery was performed under anesthesia by Avertin (250 mg/kg), and anesthetized animals were sacrificed by cervical dislocation at the end of the experiments. All experiments were performed in accordance with guidelines of the Institute for Laboratory Animal Research of Tsinghua University. The experimental procedures were approved by the Administrative Committee of Experimental Animal Care and Use of Tsinghua University, licensed by the Science and Technology Commission of Beijing Municipality (SYXK-2014-0024) and they conformed to the National Institute of Health guidelines on the ethical use of animals.

### Cell culture

Peritoneal macrophages were isolated from peritoneum by lavage using 20 ml Dulbecco's modified Eagle’s medium (DMEM) and seeded into six-well plates using one hundred million cells/well in DMEM of 10% fetal bovine serum (FBS). Non-adherent cells were removed 6 h later, whereas adherent cells were refed with DMEM of 10% FBS and allowed to recover overnight. Macrophages were treated with chlorpropamide, telmisartan and alendronate for 24 h and then pre-incubated with DMEM–10% FBS for 2 h before treatment of LPS (10 ng/ml). Macrophages were pre-treated with DuP-697 and SC-560 for 12 h before treatment with telmisartan, alendronate and chlorpropamide and then incubated with IFN-γ (10 ng/ml) for 12 h following LPS stimulation (10 ng/ml) for 6 h. The concentrations of telmisartan, alendronate and chlorpropamide treatment were determined based on previous research^62–64^, while those of the chemical probe Dub-697 and the known NSAID ibuprofen were determined according to the indications of the assay kit and previous binding studies in the literature^65–67^, respectively. Cells were harvested for subsequent analysis.

### COX fluorescent activity

Following stimulation, kidneys were harvested from mice, and macrophages from the above treatment were homogenized in 5 ml of cold phosphate-buffered saline (PBS) containing protease inhibitors and centrifuged at 10,000×*g* for 15 min at 4 °C. The supernatant was assayed by the COX fluorescent activity assay kit according to the manufacturer’s instructions.

### Human recombinant enzyme assays

The selectivity of inhibition in vitro for telmisartan, alendronate and chlorpropamide was evaluated using the recombinant human COX-1 and COX-2 enzyme assays as previously described in^68^. In particular, the recombinant enzymes were pre-incubated with various concentrations of telmisartan, alendronate and chlorpropamide for 10 min at 25 °C. Then the 10 μM arachidonic acid was added to start the reaction and allowed the process to proceed for 10 min. The reaction was terminated by diluting the reaction into buffer containing 25 μM indomethacin. The final levels of PGE 2 were measured by ELISA.

### Radioligand-based binding assays

The binding assays of Hood et al.^69^ were used to assess the direct binding activity to COX-2 by measuring the competitive binding of the radiolabeled inhibitor [^3^H] celecoxib to the target enzyme. The murine monoclonal COX-2-specific antibody was coated onto 96-well Immulon 2HB microtiter plates (Thermo Scientific, Waltham, USA) and incubated overnight at 37 °C. The coated plates were washed with Dulbecco’s PBS (D-PBS) and blocked by 10% skim milk to avoid nonspecific binding. The recombinant COX-2 enzyme binding buffer was added to plates and incubated for 2 h at 37 °C and these antibody-captured enzyme-coated plates were washed with D-PBS. To measure the competitive binding activity with celecoxib, compounds at their IC_50_ concentrations were incubated with [^3^H] celecoxib and allowed to compete for the binding to COX-2 for 2 h. After that, the incubation was halted by aspiration and washed twice with cold D-PBS. The 50 μl of 10% SDS was added into plates for 1 h at 37 °C. At last, the COX-2 bound radioligand was transferred into the liquid scintillation vial for quantitation using the liquid scintillation spectrometry.

### Real-time PCR analysis

Total RNA was extracted from the whole-cell lysates using the Trnzol-A^+^ reagent (Tiangen, Cat. no. DP421, China). Reverse transcription was performed using TIANScript RT Kit (Tiangen, Cat. no. KR104-02, China). All real-time PCR reactions were carried out on ABI ViiA 7 Real-Time System (Life Technologies, USA) using TransStart Top Green qPCR SuperMix (Transgen, Cat. no. AQ131-03, China). The formula 2^−ΔΔCt^ was used to calculate the relative expression. The expression of the housekeeping gene GAPDH was used as an internal control.

### Statistical analysis in experimental validation results

Statistical analyses were performed using GraphPad Prism software (Version 6.0). Values were presented as mean ± SD. Every analysis was performed for three independent experiments, each of which was performed with triplicates. Data were analyzed using a one-way analysis of variance, followed by a Newman–Keuls multiple comparison test. Statistical significances were calculated and indicated. *: *P* < 0.05, **: *P* < 0.01, ***: *P* < 0.001.

### Code availability

The source code of DTINet can be downloaded from https://github.com/luoyunan/DTINet.

### Data availability

The input heterogeneous network data can be downloaded from https://github.com/luoyunan/DTINet. All other data that support the results of this study are available from the corresponding author upon request.

## Electronic supplementary material

Supplementary Information

Supplementary Data 1

Supplementary Data 2

Supplementary Data 3

## References

[1] Whitebread S, Hamon J, Bojanic D, Urban L (2005). Keynote review: in vitro safety pharmacology profiling: an essential tool for successful drug development. Drug Discov. Today.

[2] Donald, B. R. *Algorithms in Structural Molecular Biology* (MIT Press, 2011).

[3] Morris GM (2009). Autodock4 and autodocktools4: automated docking with selective receptor flexibility. J. Comput. Chem..

[4] Keiser MJ (2007). Relating protein pharmacology by ligand chemistry. Nat. Biotechnol..

[5] Bleakley K, Yamanishi Y (2009). Supervised prediction of drug–target interactions using bipartite local models. Bioinformatics.

[6] Mei J-P, Kwoh C-K, Yang P, Li X-L, Zheng J (2013). Drug–target interaction prediction by learning from local information and neighbors. Bioinformatics.

[7] Xia Z, Wu L-Y, Zhou X, Wong ST (2010). Semi-supervised drug-protein interaction prediction from heterogeneous biological spaces. BMC Syst. Biol..

[8] van Laarhoven T, Nabuurs SB, Marchiori E (2011). Gaussian interaction profile kernels for predicting drug–target interaction. Bioinformatics.

[9] van Laarhoven T, Marchiori E (2013). Predicting drug-target interactions for new drug compounds using a weighted nearest neighbor profile. PLoS ONE.

[10] Wang S, Peng J (2017). Network-assisted target identification for haploinsufficiency and homozygous profiling screens. PLoS Comput. Biol..

[11] Campillos M, Kuhn M, Gavin A-C, Jensen LJ, Bork P (2008). Drug target identification using side-effect similarity. Science.

[12] Mizutani S, Pauwels E, Stoven V, Goto S, Yamanishi Y (2012). Relating drug–protein interaction network with drug side effects. Bioinformatics.

[13] Iorio F (2010). Discovery of drug mode of action and drug repositioning from transcriptional responses. Proc. Natl Acad. Sci. USA.

[14] Wang W, Yang S, Zhang X, Li J (2014). Drug repositioning by integrating target information through a heterogeneous network model. Bioinformatics.

[15] Sirota M (2011). Discovery and preclinical validation of drug indications using compendia of public gene expression data. Sci. Transl. Med..

[16] Yang, F., Xu, J. & Zeng, J. in *Pacific Symposium on Biocomputing. Pacific Symposium on Biocomputing*, 148 (NIH Public Access, 2014). PMC4730876 24297542

[17] Chen X, Liu M-X, Yan G-Y (2012). Drug–target interaction prediction by random walk on the heterogeneous network. Mol. Biosyst..

[18] Fu G (2016). Predicting drug target interactions using meta-path-based semantic network analysis. BMC Bioinformatics.

[19] Zheng, X., Ding, H., Mamitsuka, H. & Zhu, S. In *Proceedings of the 19th ACM SIGKDD international conference on Knowledge discovery and data mining*, 1025–1033 (ACM, 2013).

[20] Tong, H., Faloutsos, C. & Pan, J.-Y. In *Proceedings of the Sixth International Conference on Data Mining*, 613–622 (IEEE Computer Society, 2006).

[21] Cho, H., Berger, B. & Peng, J. in *Research in Computational Molecular Biology, Vol. 9029 of Lecture Notes in Computer Science* (ed. Przytycka, T. M.) 62–64 (Springer International Publishing, 2015). URL http: //dx.doi.org/10.1007/978-3-319-16706-0_9

[22] Cho H, Berger B, Peng J (2016). Compact integration of multi-network topology for functional analysis of genes. Cell Syst..

[23] Wang S, Cho H, Zhai C, Berger B, Peng J (2015). Exploiting ontology graph for predicting sparsely annotated gene function. Bioinformatics.

[24] Davis, J. & Goadrich, M. The relationship between precision-recall and roc curves. in *Proceedings of the 23rd International Conference on Machine learning*, 233–240 (ACM, 2006).

[25] Natarajan N, Dhillon IS (2014). Inductive matrix completion for predicting gene–disease associations. Bioinformatics.

[26] Singh-Blom UM (2013). Prediction and validation of gene-disease associations using methods inspired by social network analyses. PLoS ONE.

[27] Wu Y (2011). Evidence that clozapine directly interacts on the gabab receptor. Neuroreport.

[28] Wassef A, Baker J, Kochan LD (2003). Gaba and schizophrenia: a review of basic science and clinical studies. J. Clin. Psychopharmacol..

[29] Uefuji K, Ichikura T, Mochizuki H (2000). Cyclooxygenase-2 expression is related to prostaglandin biosynthesis and angiogenesis in human gastric cancer. Clin Cancer Res..

[30] Rao P, Knaus EE (2008). Evolution of nonsteroidal anti-inflammatory drugs (NSAIDs): cyclooxygenase (COX) inhibition and beyond. J. Pharm. Pharmaceut. Sci..

[31] Minghetti L (2004). Cyclooxygenase-2 (COX-2) in inflammatory and degenerative brain diseases. J. Neuropathol.Exp. Neurol..

[32] Kearney PM (2006). Do selective cyclo-oxygenase-2 inhibitors and traditional non-steroidal anti-inflammatory drugs increase the risk of atherothrombosis? meta-analysis of randomised trials. BMJ.

[33] Trelle S (2011). Cardiovascular safety of non-steroidal anti-inflammatory drugs: network meta-analysis. BMJ.

[34] Gosse P (2006). A review of telmisartan in the treatment of hypertension: blood pressure control in the early morning hours. Vasc. Health Risk Manag..

[35] Clarke B, Duncan L (1968). Comparison of chlorpropamide and metformin treatment on weight and blood-glucose response of uncontrolled obese diabetics. Lancet.

[36] Bianchi ML (2000). Efficacy and safety of alendronate for the treatment of osteoporosis in diffuse connective tissue diseases in children. Arthritis Rheum..

[37] DiMeglio LA, Peacock M (2006). Two-year clinical trial of oral alendronate versus intravenous pamidronate in children with osteogenesis imperfecta. J. Bone Miner. Res..

[38] Rimon G (2010). Coxibs interfere with the action of aspirin by binding tightly to one monomer of cyclooxygenase-1. Proc. Natl Acad. Sci..

[39] Vecchio AJ, Malkowski MG (2011). The structure of NS-398 bound to cyclooxygenase-2. J. Struct. Biol..

[40] Kargman S (1996). Mechanism of selective inhibition of human prostaglandin g/h synthase-1 and-2 in intact cells. Biochem. Pharmacol..

[41] Kato M, Nishida S, Kitasato H, Sakata N, Kawai S (2001). Cyclooxygenase-1 and cyclooxygenase-2 selectivity of non-steroidal anti-inflammatory drugs: investigation using human peripheral monocytes. J. Pharm. Pharmacol..

[42] Ariasnegrete S, Keller K, Chadee K (1995). Proinflammatory cytokines regulate cyclooxygenase-2 mRNA expression in human macrophages. Biochem. Biophys. Res. Commun..

[43] Mehmood S (2016). Mass spectrometry captures off-target drug binding and provides mechanistic insights into the human metalloprotease zmpste24. Nat. Chem..

[44] Franken H (2015). Thermal proteome profiling for unbiased identification of direct and indirect drug targets using multiplexed quantitative mass spectrometry. Nat. Protoc..

[45] Chernobrovkin A, Marin-Vicente C, Visa N, Zubarev RA (2015). Functional identification of target by expression proteomics (FITExP) reveals protein targets and highlights mechanisms of action of small molecule drugs. Sci. Rep..

[46] Savitski MM (2014). Tracking cancer drugs in living cells by thermal profiling of the proteome. Science.

[47] Guney E, Menche J, Vidal M, Barábasi A-L (2016). Network-based in silico drug efficacy screening. Nat. Commun..

[48] Cheng F, Zhao J, Fooksa M, Zhao ZA (2016). A network-based drug repositioning infrastructure for precision cancer medicine through targeting significantly mutated genes in the human cancer genomes. J. Am. Med. Inform. Assoc..

[49] Benson SC (2004). Identification of telmisartan as a unique angiotensin ii receptor antagonist with selective pparγ–modulating activity. Hypertension.

[50] Sato K (2014). Telmisartan ameliorates inflammatory responses in SHR-SR after tMCAO. J. Stroke Cerebrovasc. Dis..

[51] Lanz TV (2010). Angiotensin ii sustains brain inflammation in mice via tgf-*β*. J. Clin. Invest..

[52] Giles FJ (2002). The emerging role of angiogenesis inhibitors in hematologic malignancies. Oncology (Williston Park).

[53] Lubran MM (1989). Hematologic side effects of drugs. Ann. Clin. Lab. Sci..

[54] Töyräs A, Ollikainen J, Taskinen M, Mönkkönen J (2003). Inhibition of mevalonate pathway is involved in alendronate-induced cell growth inhibition, but not in cytokine secretion from macrophages in vitro. Eur. J. Pharmaceut. Sci..

[55] Bengio Y, Courville A, Vincent P (2013). Representation learning: a review and new perspectives. IEEE Trans. Pattern Anal. Mach. Intell..

[56] Knox C (2011). Drugbank 3.0: a comprehensive resource for ‘omics’ research on drugs. Nucleic Acids Res..

[57] Prasad TSK (2009). Human protein reference database—2009 update. Nucleic Acids Res..

[58] Davis AP (2013). The comparative toxicogenomics database: update 2013. Nucleic Acids Res..

[59] Kuhn, M., Campillos, M., Letunic, I., Jensen, L. J. & Bork, P. A side effect resource to capture phenotypic effects of drugs. **6**, 343 (2009). 10.1038/msb.2009.98 PMC2824526 20087340

[60] Berman HM (2000). The protein data bank. Nucleic Acids Res..

[61] Irwin JJ, Sterling T, Mysinger MM, Bolstad ES, Coleman RG (2012). Zinc: a free tool to discover chemistry for biology. J. Chem. Inf. Model..

[62] Liu J (2014). Differential effects of angiotensin II receptor blockers on a*β* generation. Neurosci. Lett..

[63] Tsubaki M (2012). Bisphosphonate-and statin-induced enhancement of OPG expression and inhibition of CD9, M-CSF, and RANKL expressions via inhibition of the Ras/MEK/ERK pathway and activation of p38MAPK in mouse bone marrow stromal cell line ST2. Mol. Cell. Endocrinol..

[64] Durr JA, Hensen J, Ehnis T, Blankenship MS (2000). Chlorpropamide upregulates antidiuretic hormone receptors and unmasks constitutive receptor signaling. Am. J. Physiol. Renal Physiol..

[65] Aeberhard EE (1995). Nonsteroidal anti-inflammatory drugs inhibit expression of the inducible nitric oxide synthase gene. Biochem. Biophys. Res. Commun..

[66] Rosenstock M, Danon A, Rimon G (1999). PGHS-2 inhibitors, NS-398 and DuP-697, attenuate the inhibition of PGHS-1 by aspirin and indomethacin without altering its activity. Biochim. Biophys. Acta.

[67] Stuhlmeier KM, Li H, Kao JJ (1999). Ibuprofen: new explanation for an old phenomenon. Biochem. Pharmacol..

[68] Gierse JK (1995). Expression and selective inhibition of the constitutive and inducible forms of human cyclo-oxygenase. Biochem J..

[69] Hood WF (2003). Characterization of celecoxib and valdecoxib binding to cyclooxygenase. Mol. Pharmacol..

[70] Schrödinger, L. L. C. The PyMOL molecular graphics system, version 1.8 (2015).

