# Supplementary material for: A network integration approach for drug-target interaction prediction and computational drug repositioning from heterogeneous information
Source: Nat Commun. 2017 Sep 18;8:573. doi: 10.1038/s41467-017-00680-8 (PMC5603535; doi:10.1038/s41467-017-00680-8)
Supplement: Supplementary file 1 — Supplementary Information [file 41467_2017_680_MOESM1_ESM.pdf]

Description of Supplementary Files

File name: Supplementary Information
Description: Supplementary figures, supplementary tables, supplementary note and supplementary references.

File name: Supplementary Data 1
Description: The list of top 150 novel drug-target interactions predicted by DTINet, which was trained based all on drugs and targets that have at least one known interacting pair. Known drug-target pairs (corresponding to those non-zero entries in the drug-target interaction matrix) and novel predicted DTIs that share homologous proteins (with sequence identity scores >40%) with known DTIs were excluded from the list. Also available on https://github.com/luoyunan/DTINet.

File name: Supplementary Data 2
Description: The entire list of novel drug-target interactions predicted by DTINet, which was trained based on all drugs and targets that have at least one known interacting pair. Also available on https://github.com/luoyunan/DTINet.

File name: Supplementary Data 3
Description: Examples of the novel predictions which can be supported by the previous known evidence in the literature. Also available on https://github.com/luoyunan/DTINet.

Supplementary Note 1

Compact feature learning for drugs and targets

DTINet applies diffusion component analysis (DCA) [1], a recently developed algorithm that combines network diffusion (i.e., random walk with restart) with dimensionality reduction, to learn the low-dimensional vector representations of the drug and target features that capture the intrinsic topological properties of a heterogeneous network. DCA has recently been generalized into Mashup, a new method for integrating multiple heterogeneous interactomes [2].

Random walk with restart revisited. Random walk with restart (RWR), a network diffusion algorithm, has been extensively applied to analyze the complex biological network data [3, 4, 5, 6, 7]. Different from conventional random walk methods, RWR introduces a pre-defined restart probability at the initial node for every iteration, which can take into consideration both local and global topological connectivity patterns within the network to fully exploit the underlying direct or indirect relations between nodes. Formally, let $\mathbf{A}$ denote the weighted adjacency matrix of a molecular interaction network with n drugs (or targets). We also define another matrix $\mathbf{B}$, in which each element $\mathbf{B}_{i,j}$ describes the probability of a transition from node i to node j, that is,

$$\mathbf{B}_{i,j} = \frac{\mathbf{A}i,j}{\sum_{j'} \mathbf{A}_{i,j'}} \tag{1}$$

Next, let $\mathbf{s}_i^t$ be an n-dimensional distribution vector in which each element stores the probability of a node being visited from node i after t iterations in the random walk process. Then RWR from node i can be defined as:

$$\mathbf{s}_i^{t+1} = (1 - p_r)\mathbf{s}_i^t\mathbf{B} + p_r\mathbf{e}_i, \tag{2}$$

where $\mathbf{e}_i$ stands for an n-dimensional standard basis vector with $\mathbf{e}_i(i) = 1$ and $\mathbf{e}_i(j) = 0$, $\forall j \neq i$, and p_r stands for the pre-defined restart probability, which actually controls the relative influence between local and global topological information in the diffusion process, with higher values emphasizing more on the local structures in the network. At some fixed point of the iterating process, we can obtain a stationary distribution $\mathbf{s}_i^\infty$ of RWR, which we refer to as the "diffusion state" $\mathbf{s}_i$ for node i (i.e., $\mathbf{s}_i = \mathbf{s}_i^\infty$), being consistent with the notation of previous work [1]. Intuitively, the jth element of diffusion state, denoted by $\mathbf{s}_{ij}$, repre-

sents the probability of RWR starting node i and ending up at node j in equilibrium. When two nodes have similar diffusion states, it generally implies that they have similar positions with respect to other nodes in the network, and thus probably share similar functions.

The dimensionality reduction framework. The diffusion states resulting from the aforementioned RWR process may not be entirely accurate, partially due to the low-quality and high-dimensionality of biological data. A small number of missing or fake interactions in the network can significantly affect the results of the diffusion process [8]. Moreover, it is generally inconvenient to directly use the high dimensionality of the diffusion states for the topological features, especially for our heterogeneous network based prediction task. To address this issue, DTINet employs a dimensionality reduction scheme, called diffusion component analysis (DCA), to reduce the dimensionality of feature space and capture those important topological features from the diffusion states. With the goal of denoise and dimensionality reduction, DCA approximates each diffusion state $\mathbf{s}_i$ with a multinomial logistic model based on a latent vector representation whose dimensionality is much lower than that of the original n-dimensional vector representing the diffusion states. Specifically, the probability assigned to node j in the diffusion state of node i is now modeled as

$$\hat{\mathbf{s}}_{ij} = \frac{\exp\left(\mathbf{w}_i^T \mathbf{x}_j\right)}{\sum_{j'} \exp\left(\mathbf{w}_i^T \mathbf{x}_{j'}\right)} \tag{3}$$

where $\forall i$, $\mathbf{x}_i, \mathbf{w}_i \in \mathbb{R}^d$ for $d \ll n$. We refer to $\mathbf{w}_i$ as the *context feature* and $\mathbf{x}_i$ as the *node feature* for node i, both describing the topological properties of the network. If $\mathbf{x}_i$ and $\mathbf{w}_j$ point to a similar direction and thus have a large inner product, it is likely that node j is frequently visited in a random walk starting from node i. DCA takes a set of the observed diffusion states $S = \{\mathbf{s}_1, \ldots, \mathbf{s}_n\}$ as input and optimizes over $\mathbf{w}$ and $\mathbf{x}$ for all nodes, using the Kullback-Leibler (KL) divergence (also called relative entropy) to guide the optimization, that is,

$$\min_{\mathbf{w},\mathbf{x}} C(\mathbf{s}, \hat{\mathbf{s}}) = \frac{1}{n} \sum_{i=1}^{n} D_{KL}(\mathbf{s}_i \parallel \hat{\mathbf{s}}_i), \tag{4}$$

where $D_{KL}(\cdot \parallel \cdot)$ denotes the KL-divergence between two distributions. The DCA framework uses a standard quasi-Newton method L-BFGS [9] to solve this optimization problem.

Integration of heterogeneous network information. The above dimensionality reduction framework can be naturally extended to integrate multiple network data from heterogeneous sources. Given K similarity

networks in a heterogeneous framework constructed from diverse information (see "Construction of similarity networks" in Supplementary Note 1), DCA first performs RWR on individual networks separately and then obtains the network-specific diffusion states $\mathbf{s}_i^{(k)}$ for each node i in every network k. After that, it also constructs a multinomial logistic distribution to model the diffusion states:

$$\hat{\mathbf{s}}_{ij}^{(k)} = \frac{\exp\left(\mathbf{w}_i^{(k)T}\mathbf{x}_j\right)}{\sum_{j'}\exp\left(\mathbf{w}_i^{(k)T}\mathbf{x}_{j'}\right)}, \tag{5}$$

where each node i is assigned with a network-specific vector representation $\mathbf{w}_i^{(k)}$, which represents the context feature of node i in network k, and the node feature vectors $\mathbf{x_i}$ are allowed to be shared globally across all K networks. Finally, DCA optimizes the following objective function,

$$\min_{\mathbf{w},\mathbf{x}} C(\mathbf{s},\hat{\mathbf{s}}) = \sum_{k=1}^{K}\frac{1}{n}\sum_{i=1}^{n} D_{KL}\left(\mathbf{s}_i^{(k)} \parallel \hat{\mathbf{s}}_i^{(k)}\right), \tag{6}$$

which can also be solved by the quasi-Newton L-BFGS method [9]. Although the divergence terms of individual networks are given equal weights in the above objective function, it is possible to weight them differently to emphasize the relative importance of individual networks.

To make the DCA framework more scalable to large biological networks, DTINet employs a variant of DCA, called clusDCA [10], which uses an alternative objective function that can be optimized efficiently based on singular value decomposition (SVD) (see "The optimization process of DCA" in Supplementary Note 1). Briefly, for each drug or protein, clusDCA is able to learn a low-dimensional vector representation that corresponds to a solution minimizing the difference between the observed diffusion states and the model distribution under the $L2$-norm in log space [10].

In our tests, we observed stable performance of DTINet for different values of the restart probability p_r between 0.5 and 0.8 (Supplementary Figure 9a). For all the test results shown in the Results section, the restart probability p_r was set to 0.8. After dimensionality reduction, we learned an f_d-dimensional vector for drugs and an f_t-dimensional vectors for targets. We observed robust results over a wide range of choices for the f_d and f_t parameters (Supplementary Figure 8). In the tests, we set $f_d = 100$ and $f_t = 400$, which were equal to 10-20% of the dimensionality of the original vectors describing the diffusion states.

The optimization process of DCA

For simplicity, here we only show the optimization process of DCA for a single input network. The optimization of DCA with multiple networks is a simple extension. To optimize the following objective function of DCA,

$$\min_{\mathbf{w},\mathbf{x}} C(\mathbf{s},\hat{\mathbf{s}}) = \frac{1}{n}\sum_{i=1}^{n} D_{KL}(\mathbf{s}_i \parallel \hat{\mathbf{s}}_i), \tag{7}$$

we first express the formula in terms of $\mathbf{w}$ and $\mathbf{x}$ based on the definition of KL-divergence and $\hat{\mathbf{s}}$, that is,

$$C(\mathbf{s},\hat{\mathbf{s}}) = \frac{1}{n}\sum_{i=1}^{n}\left[H(\mathbf{s}_i) - \sum_{j=1}^{n}\mathbf{s}_{ij}\left(\mathbf{w}_i^T\mathbf{x}_j - \log\left(\sum_{j'=1}^{n}\exp\{\mathbf{w}_i^T\mathbf{x}_{j'}\}\right)\right)\right], \tag{8}$$

where $H(\cdot)$ denotes the entropy. Then we compute the gradients of this objective with respect to the parameters $\mathbf{w}$ and $\mathbf{x}$, respectively,

$$\nabla_{\mathbf{w}_i} C(\mathbf{s},\hat{\mathbf{s}}) = \frac{1}{n}\sum_{j=1}^{n}(\hat{\mathbf{s}}_{ij} - \mathbf{s}_{ij})\mathbf{x}_j, \tag{9}$$

$$\nabla_{\mathbf{x}_i} C(\mathbf{s},\hat{\mathbf{s}}) = \frac{1}{n}\sum_{j=1}^{n}(\hat{\mathbf{s}}_{ji} - \mathbf{s}_{ji})\mathbf{w}_j. \tag{10}$$

This objective function can be solved using a standard quasi-Newton L-BFGS method to find the low-dimensional vector representations $\mathbf{w}$ and $\mathbf{x}$. Throughout our tests, the vectors $\mathbf{w}$ and $\mathbf{x}$ were initialized with uniform random values in $[-0.05, 0.05]$.

As mentioned in the main text, to make the DCA framework more scalable to large biological networks, we use a more efficient matrix factorization based approach to decompose the diffusion states and obtain their low-dimensional vector representations. Based on the definition of $\hat{\mathbf{s}}_{ij}$, we have

$$\log \hat{\mathbf{s}}_{ij} = \mathbf{x}_i^T\mathbf{w}_j - \log\sum_{j'}\exp\{\mathbf{w}_i^T\mathbf{x}_{j'}\}. \tag{11}$$

The first term in the above equation corresponds to the low-dimensional approximation of $\hat{\mathbf{s}}_{ij}$, and the second term is a normalization factor, which ensures that $\hat{\mathbf{s}}_i$ is a well defined distribution. We relax the constraint that the entries in $\hat{\mathbf{s}}_i$ must sum to one by dropping the second term, that is

$$\log \hat{\mathbf{s}}_{ij} = \mathbf{x}_i^T\mathbf{w}_j. \tag{12}$$

In addition, instead of minimizing the relative entropy between the original and approximated diffusion

states, we use the sum of squared errors as the objective function:

$$\min_{\mathbf{w},\mathbf{x}} C(\mathbf{s}, \hat{\mathbf{s}}) = \sum_{i=1}^{n}\sum_{j=1}^{n}(\mathbf{x}_i^T \mathbf{w}_j - \log \hat{\mathbf{s}}_{ij})^2. \tag{13}$$

This resulting objective function can be optimized by singular value decomposition (SVD). To avoid taking the logarithm of zero, we add a small positive constant $\frac{1}{n}$ to $\mathbf{s}_{ij}$ and compute the logarithm diffusion state matrix $\mathbf{L}$ as:

$$\mathbf{L} = \log(\mathbf{S} + \mathbf{Q}) - \log(\mathbf{Q}), \tag{14}$$

where $\mathbf{Q} \in \mathbb{R}^{n\times n}$ with $\mathbf{Q}_{ij} = \frac{1}{n}$, $\forall i, j$, and $\mathbf{S} \in \mathbb{R}^{n\times n}$ is the concatenation of $\mathbf{s}_1, \ldots, \mathbf{s}_n$. With SVD, we decompose $\mathbf{L}$ into three matrices:

$$\mathbf{L} = \mathbf{U}\boldsymbol{\Sigma}\mathbf{V}^T. \tag{15}$$

To obtain the low-dimensional vectors $\mathbf{w}_j$ and $\mathbf{x}_i$ of d dimensions, we simply choose the first d singular vectors in $\mathbf{U}_d$, $\mathbf{V}_d$ and the first d singular values in $\boldsymbol{\Sigma}_d$. More precisely, let $\mathbf{X} = [\mathbf{x}_1, \ldots, \mathbf{x}_n]^T$ denote a matrix where each row represents the corresponding low-dimensional feature vector representation of each node in the network, and let $\mathbf{W} = [\mathbf{w}_1, \ldots, \mathbf{w}_n]^T$ denote a matrix where each row represents the corresponding vector of the context features. Then, $\mathbf{X}$ and $\mathbf{W}$ can be computed as:

$$\mathbf{X} = \mathbf{U}_d\boldsymbol{\Sigma}_d^{1/2}, \quad \mathbf{W} = \mathbf{V}_d\boldsymbol{\Sigma}_d^{1/2}. \tag{16}$$

To integrate heterogeneous network data, we extend the above single-network DCA to a multiple-network case. More specifically, let $\mathbb{L} = \{\mathbf{L}^1, \ldots, \mathbf{L}^K\}$ be the set of logarithm diffusion state matrices based on the set of diffusion states $\mathbb{S} = \{\mathbf{S}^1, \ldots, \mathbf{S}^K\}$ from K input networks. Then, we optimize the following objective function:

$$\min_{\mathbf{w},\mathbf{x}} C(\mathbb{S}, \widehat{\mathbb{S}}) = \sum_{i=1}^{n}\sum_{j=1}^{n}\sum_{r=1}^{K}(\mathbf{x}_i^T \mathbf{w}_j^r - \log \hat{\mathbf{s}}_{ij}^r)^2, \tag{17}$$

where we assign a network-specific feature $\mathbf{w}_i^r$ for each node i in network r, and the node features $\mathbf{x}_i$ are shared across all K networks. This objective function can also be optimized by SVD.

Projection from drug space onto target space

We use the low-dimensional vector representations of both drug and protein features obtained from compact feature learning to predict new drug-target interactions. Based on the intuition that geometric prox-

imity in the feature vector space may reflect the functional relevance, we apply a matrix completion approach [11] to obtain a projection matrix that maps the low-dimensional feature vectors from drug space onto protein space, such that the projected feature vectors of drugs are geometrically close to the vectors of their known interacting proteins.

Formally, we use $\mathbf{X} = [\mathbf{x}_1, \ldots, \mathbf{x}_{N_d}]^T$, $\mathbf{x}_i \in \mathbb{R}^{f_d}, i = 1, \ldots, N_d$ to denote the matrix representation of the drug features (i.e., each row i represents the corresponding feature vector of drug i), and $\mathbf{Y} = [\mathbf{y}_1, \ldots, \mathbf{y}_{N_t}]^T$, $\mathbf{y}_i \in \mathbb{R}^{f_t}, i = 1, \ldots, N_t$, to denote the matrix representation of the protein features (i.e., each row i represents the corresponding feature vector of protien i), where N_d and N_t stand for the numbers of drugs and proteins, respectively. Let $\mathbf{P}$ be a drug-target interaction matrix, where each entry $\mathbf{P}_{ij} = 1$ if drug i is known to interact with protein j, and $\mathbf{P}_{ij} = 0$ otherwise. We set up a bilinear function to learn the projection matrix $\mathbf{Z}$ between drug space and target space to predict the unknown drug-target interactions in $\mathbf{P}$ (i.e., those zero-valued entries). In particular, the bilinear function is formulated as:

$$\mathbf{X}\mathbf{Z}\mathbf{Y}^T \approx \mathbf{P}, \tag{18}$$

where $\mathbf{P} \in \mathbb{R}^{N_d \times N_t}$ stands for the drug-target interaction matrix, $\mathbf{X} \in \mathbb{R}^{N_d \times f_d}$, $\mathbf{Y} \in \mathbb{R}^{N_t \times f_t}$ are obtained from the compact feature learning stage (i.e., the network diffusion and dimensionality reduction processes), and $\mathbf{Z} \in \mathbb{R}^{f_d \times f_t}$ is the projection matrix to be learned. We then use the formula below to measure the likelihood of the pairwise interaction score between drug i and protein j:

$$\mathrm{score}(i, j) = \mathbf{x}_i \mathbf{Z} \mathbf{y}_j^T, \tag{19}$$

where a larger $\mathrm{score}(i, j)$ suggests that drug i is more likely to interact with protein j.

Although the projection matrix $\mathbf{Z}$ is of dimension $f_d \times f_t$, there typically exist significant correlations between those feature vectors of drugs or proteins that are geometrically close in space, which can thus greatly reduce the number of effective parameters required to model drug-target interactions. To take into account this issue, we impose a low-rank constraint on $\mathbf{Z}$ to learn only a small number of latent factors, by considering a low-rank decomposition of the form $\mathbf{Z} = \mathbf{G}\mathbf{H}^T$, where $\mathbf{G} \in \mathbb{R}^{f_d \times f_k}$ and $\mathbf{H} \in \mathbb{R}^{f_t \times f_k}$. This low-rank constraint not only alleviates the overfitting problem but also computationally benefits the optimization process [12]. The optimization problem with such a low-rank constraint on the original projection

matrix $\mathbf{Z}$ is NP-hard to solve. A standard relaxation of the low-rank constraint is to minimize the trace norm (i.e., sum of singular values) of the matrix $\mathbf{Z} = \mathbf{G}\mathbf{H}^T$, which is equivalent to minimize the Frobenius norms $\frac{1}{2}(\|\mathbf{G}\|_F^2 + \|\mathbf{H}\|_F^2)$. Therefore, factoring $\mathbf{Z}$ into $\mathbf{G}$ and $\mathbf{H}$ can be accomplished by solving the following optimization problem:

$$\min_{\mathbf{G},\mathbf{H}} \sum_{(i,j)} \|\mathbf{P}_{ij} - \mathbf{x}_i \mathbf{G}\mathbf{H}^T \mathbf{y}_j^T\|_2^2 + \frac{\lambda}{2}(\|\mathbf{G}\|_F^2 + \|\mathbf{H}\|_F^2), \tag{20}$$

where λ is a regularization parameter, which controls the tradeoff between the minimization of the squared loss on known interaction pairs (i, j) and the Frobenius norms. The optimization problem can be solved by alternating minimization [11]. We evaluated the prediction performance of DTINet with respect to different choices of the latent dimensionality parameter f_k and observed stable performance of DTINet over a wide range values of this parameter (Supplementary Figure 9b). In our test, we set the value of the latent dimensionality parameter to $f_k = 50$, which was roughly equal to 50% of the dimension of the feature vectors of drugs, and 10% of the dimension of the feature vectors of proteins. The performance of DTINet was also robust to different choices of the regularization parameter λ. We observed that the performance was not influenced much when varying λ from 10^{-3} to 10^2 (Supplementary Figure 9c). In our test, we did not fine-tune the value of λ and simply set $\lambda = 1$. In principle, we could always further improve the performance by carefully tuning these parameters using an inner-loop cross-validation on training data.

Time complexity of DTINet

When learning the low-dimensional representations of nodes in a heterogeneous network, performing the random walk with restart on each single network takes time complexity $O(n^3)$, where n is the number of nodes in the network. Next, the SVD operation on the matrix of diffusion states takes $O(Kn^3)$ time, where K stands for the total number of individual networks in the heterogeneous framework. Therefore, the running time for learning the compact representations of drugs and targets is $O(K_d N_d^3)$ and $O(K_t N_t^3)$, respectively, where K_d and K_t stand for the total numbers of similarity networks for drugs and targets, respectively, and N_d and N_t stand for the total numbers of drugs and targets, respectively. The matrix completion step for learning the projection matrix takes $O((q + N_d f_d + N_t f_t) f_k^2)$, where q stands for the number of non-zero entries in the known drug-target interaction matrix, f_d and f_t stand for the dimensions

of the low-dimensional vector representations of drugs and targets, respectively, and f_k stands for the latent rank parameter of the matrix completion [11]. Thus, the overall time complexity of DTINet is $O(K_d N_d^3 + K_t N_t^3 + (q + N_d f_d + N_t f_t) f_k^2)$. In practice, K_d, K_t and f_k are usually small and can be regarded as constants.

Construction of similarity networks

For the input homogeneous interaction networks (e.g., drug-drug interaction network), we compute the "diffusion state" of each drug or target by directly running the RWR algorithm on each of these networks. For the association networks, i.e., drug-side-effect, drug-disease, and protein-disease association networks, we construct the corresponding similarity networks based on the Jaccard similarity coefficient and then run the RWR process on these similarity networks. Jaccard similarity is a common statistic used to characterize the similarity between two sets of objects. Taking the drug-side-effect association network as an example, we use the following formula to measure the similarity between drug i and drug j:

$$\mathbf{S}(i,j) = \frac{|SE_i \cap SE_j|}{|SE_i \cup SE_j|}, \tag{21}$$

where SE_i denotes the set of side-effects of drug i. Then we run the RWR procedure on this similarity network to obtain the diffusion states of drugs. In the same manner, we can construct the similarity networks of proteins.

In addition to the above interaction or association-based similarity networks, we construct a drug similarity network based on the chemical structures of drugs, in which the similarity score between a pair of two drugs is calculated using the Tanimoto coefficient [13] using the product-graphs of their chemical structures. We also construct a protein similarity network based on genome sequences, in which the similarity score between a pair of two proteins is computed using the Smith-Waterman score [14] based on their primary sequences.

Overall, we construct four similarity networks for drugs, based on (i) drug-drug interactions, (ii) drug-disease associations, (iii) drug-side-effect associations, and (iv) chemical structures. Similarly, we construct three similarity networks for proteins, based on (i) protein-protein interactions, (ii) protein-disease associations, and (iii) genome sequences. With these similarity networks, we can learn the low-dimensional feature vector representations of drugs and proteins, by first performing diffusion separately on individual

networks and then jointly optimizing the feature vectors under the compact feature learning framework.

Baseline Methods

We compare our method against four previously-proposed methods, including the bipartite local models (BLMNII), the Laplacian regularized least square (NetLapRLS), the heterogeneous network model (HNM) and the collaborative matrix factorization (CMF). We briefly describe these methods below.

1. **Bipartite local model with neighbor-based interaction-profile inferring (BLMNII)** [15]: This method is a combination of the bipartite local model (BLM) and the neighbor-based interaction-profile inferring (NII). The BLM framswork models the drug-target interaction prediction task as a binary classification problem in a bipartite graph. Suppose that we want to predict whether drug d_i interacts with target t_j. The BLM method first focuses on drug d_i and assigns a label $+1$ to all the known targets that interact with drug d_i, and -1 otherwise. Then BLM uses the protein similarity matrix as a kernel matrix to train a support vector machine (SVM). Such a process is also performed in a reverse way, that is, BLM also labels each known drug by whether it interacts with target t_j or not, and then trains an SVM based on the drug similarity matrix. The final prediction of whether drug d_i interacts with target t_j is then derived based on the average prediction score from both directions. The NII procedure incorporates the neighbors interaction profiles into the BLM method to train the model and enable the prediction for new drugs or targets.

2. **Laplacian regularized least square (NetLapRLS)** [16]: This method employs a semi-supervised learning algorithm, i.e., Laplacian regularized least square, for DTI prediction, which utilizes available labeled data of DTI pairs and incorporates similarity and interaction kernels to improve the prediction. NetLapRLS attempts to estimate the interaction scores $\mathbf{F}_d$ and $\mathbf{F}_t$ based on the drug and protein domains, respectively. For example, the interaction scores $\mathbf{F}_d$ are obtained by minimizing the squared loss between the known DTI matrix $\mathbf{P}$ and $\mathbf{F}_d$ with a regularized term of $\mathbf{F}_d$ and $\mathbf{S}_d$, where $\mathbf{S}_d$ is the similarity network of drugs. The final prediction $\mathbf{F}$ is obtained by averaging the results derived from both $\mathbf{F}_d$ and $\mathbf{F}_t$.

3. **Heterogeneous network model (HNM)** [17]: This method builds a three-layer heterogeneous network consisting of three types of nodes: drug, target and disease nodes. Then it iteratively propagates interaction or association information in the heterogenous network using random walk with restart. The iterative updating rule is given by

$$\mathbf{W}_{td}^{k+1} = \alpha \mathbf{W}_{td}^{k} \times (\mathbf{W}_{dd} \times \mathbf{W}_{ds}^{k} \times \mathbf{W}_{ss} \times \mathbf{W}_{ds}^{kT}) + (1-\alpha)\mathbf{W}_{td}^{0}, \quad (22)$$

$$\mathbf{W}_{ds}^{k+1} = \alpha (\mathbf{W}_{td}^{kT} \times \mathbf{W}_{tt} \times \mathbf{W}_{td}^{k} \times \mathbf{W}_{dd}) \times \mathbf{W}_{ds}^{k} + (1-\alpha)\mathbf{W}_{ds}^{0}, \quad (23)$$

where $\mathbf{W}_{td}^{k}$ and $\mathbf{W}_{ds}^{k}$ stand for the weights on the target-drug and drug-disease association links in the kth iteration, respectively; $\mathbf{W}_{td}^{0}$ and $\mathbf{W}_{ds}^{0}$ represent the target-drug and drug-disease association matrices defined by the input data; $\mathbf{W}_{dd}$ stores both drug interaction and similarity information, which is basically computed from the averaging result derived from both of the drug-drug interaction and drug-drug similarity matrices; $\mathbf{W}_{ss}$ represents the disease-disease similarity matrix; and $\mathbf{W}_{tt}$ represents the protein-protein interaction matrix derived from the input data. The final DTI prediction scores are obtained from matrix $\mathbf{W}_{td}$ after convergence.

4. **Collaborative Matrix factorization (CMF)** [18]: This method learns the feature vector matrices $\mathbf{X}$ and $\mathbf{Y}$ for drugs and targets, respectively, by minimize the following objective function:

$$\min_{\mathbf{X},\mathbf{Y}} \|\mathbf{P} - \mathbf{X}\mathbf{Y}^T\|_F^2 + \lambda_m(\|\mathbf{X}\|_F^2 + \|\mathbf{Y}\|_F^2) + \lambda_d\|\mathbf{S}_d - \mathbf{X}\mathbf{X}^T\|_F^2 + \lambda_t\|\mathbf{S}_t - \mathbf{Y}\mathbf{Y}^T\|_F^2, \quad (24)$$

where S_d and S_t represent the drug and target similarity matrices, respectively, and λ_m, λ_d and λ_t represent the regularization coefficients.

Here, HNM and CMF are designed to integrate heterogeneous information, while BLMNII and NetLapRLS mainly focus on solving the DTI prediction problem on a single network. To make a fair comparison, we first summarized our heterogeneous network into a single network for both BLMNII and NetLapRLS, using the following integration process. In particular, we combined multiple networks into a single network by assigning the edge weight $p_{i,j} = 1 - \prod_k (1 - p_{i,j}^{(k)})$, where $p_{i,j}^{(k)} \in [0,1]$ is the interaction probability or similarity between node i and node j in network $k \in \{1, 2, \ldots, K\}$, where K stands for the total number of networks.

Supplementary References

[1] Cho, H., Berger, B. & Peng, J. Diffusion component analysis: Unraveling functional topology in biological networks. In Przytycka, T. M. (ed.) *Research in Computational Molecular Biology*, vol. 9029 of *Lecture Notes in Computer Science*, 62–64 (Springer International Publishing, 2015). URL `http://dx.doi.org/10.1007/978-3-319-16706-0_9`.

[2] Cho, H., Berger, B. & Peng, J. Compact integration of multi-network topology for functional analysis of genes. *Cell Systems* **3**, 540–548 (2016).

[3] Cao, M. *et al.* New directions for diffusion-based network prediction of protein function: incorporating pathways with confidence. *Bioinformatics* **30**, i219–i227 (2014).

[4] Köhler, S., Bauer, S., Horn, D. & Robinson, P. N. Walking the interactome for prioritization of candidate disease genes. *The American Journal of Human Genetics* **82**, 949–958 (2008).

[5] Navlakha, S. & Kingsford, C. The power of protein interaction networks for associating genes with diseases. *Bioinformatics* **26**, 1057–1063 (2010).

[6] Liao, C.-S., Lu, K., Baym, M., Singh, R. & Berger, B. Isorankn: spectral methods for global alignment of multiple protein networks. *Bioinformatics* **25**, i253–i258 (2009).

[7] Chen, X., Liu, M.-X. & Yan, G.-Y. Drug–target interaction prediction by random walk on the heterogeneous network. *Molecular BioSystems* **8**, 1970–1978 (2012).

[8] Kim, M. & Leskovec, J. The network completion problem: Inferring missing nodes and edges in networks. In *SDM*, vol. 11, 47–58 (SIAM, 2011).

[9] Zhu, C., Byrd, R. H., Lu, P. & Nocedal, J. Algorithm 778: L-bfgs-b: Fortran subroutines for large-scale bound-constrained optimization. *ACM Transactions on Mathematical Software (TOMS)* **23**, 550–560 (1997).

[10] Wang, S., Cho, H., Zhai, C., Berger, B. & Peng, J. Exploiting ontology graph for predicting sparsely annotated gene function. In *ISMB/ECCB* (2015).

[11] Natarajan, N. & Dhillon, I. S. Inductive matrix completion for predicting gene–disease associations. *Bioinformatics* **30**, i60–i68 (2014).

[12] Yu, H.-F., Jain, P., Kar, P. & Dhillon, I. S. Large-scale multi-label learning with missing labels. In *ICML* (2014).

[13] Hattori, M., Okuno, Y., Goto, S. & Kanehisa, M. Development of a chemical structure comparison method for integrated analysis of chemical and genomic information in the metabolic pathways. *Journal of the American Chemical Society* **125**, 11853–11865 (2003).

[14] Smith, T. F. & Waterman, M. S. Identification of common molecular subsequences. *Journal of molecular biology* **147**, 195–197 (1981).

[15] Mei, J.-P., Kwoh, C.-K., Yang, P., Li, X.-L. & Zheng, J. Drug–target interaction prediction by learning from local information and neighbors. *Bioinformatics* **29**, 238–245 (2013).

[16] Xia, Z., Wu, L.-Y., Zhou, X. & Wong, S. T. Semi-supervised drug-protein interaction prediction from heterogeneous biological spaces. *BMC systems biology* **4**, S6 (2010).

[17] Wang, W., Yang, S., Zhang, X. & Li, J. Drug repositioning by integrating target information through a heterogeneous network model. *Bioinformatics* **30**, 2923–2930 (2014).

[18] Zheng, X., Ding, H., Mamitsuka, H. & Zhu, S. Collaborative matrix factorization with multiple similarities for predicting drug-target interactions. In *KDD* (2013).

Type of node	Count
Drug	708
Protein	1,512
Disease	5,603
Side-effect	4,192
Total	12,015

Supplementary Table 1: The number of nodes of individual types in the constructed heterogeneous network.

Type of edge	Count
Drug-Protein	1,923
Drug-Drug	10,036
Drug-Disease	199,214
Drug-Side-effect	80,164
Protein-Protein	7,363
Protein-Disease	1,596,745
Total	1,895,445

Supplementary Table 2: The size of individual interaction or association matrices in the constructed heterogeneous network.

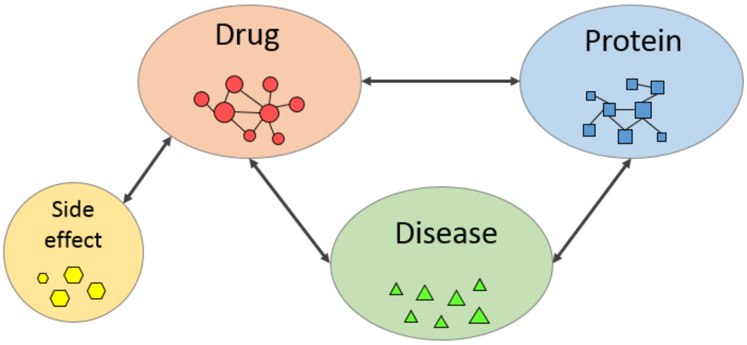

Supplementary Figure 1: The schema of the heterogeneous network constructed based on diverse data sources. Each edge between different types of nodes represents the pairwise interactions or associations between two nodes of the corresponding types (e.g., drug-protein interaction or protein-disease association). Each edge of a homogeneous drug (or protein) network represents the similarity between two drugs (or proteins) or their interaction.

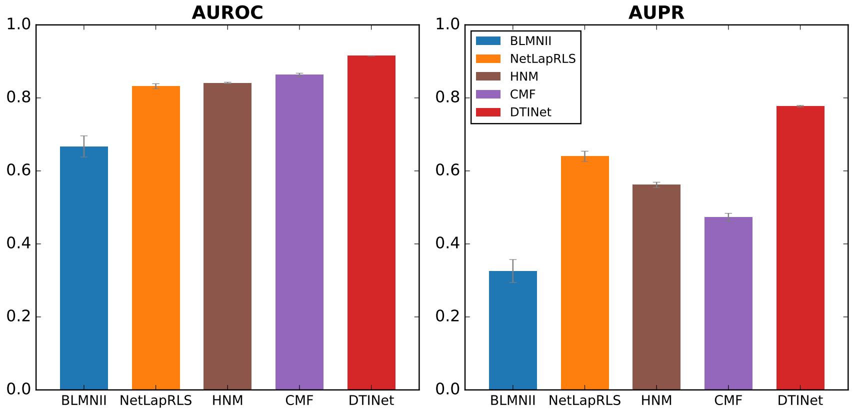

(a)

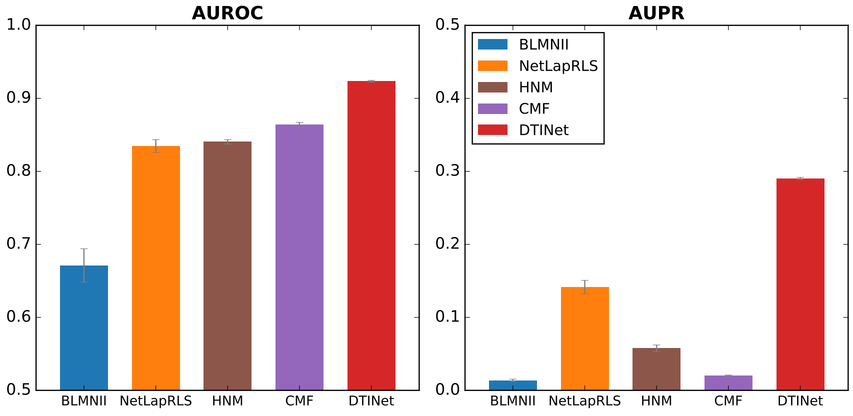

(b)

Supplementary Figure 2: Performance comparisons between DTINet and other state-of-the-art methods on skewed datasets. (a) The number of randomly chosen non-interacting drug-target pairs (i.e., negative samples) was 10 times more than the number of known interacting drug-target pairs (i.e., positive samples). (b) The negative set include all the remaining non-interacting drug-target pairs that were not in the training data. Here, during the training process, a randomly chosen subset of 90% known interactions and a matching number of non-interacting pairs were used to train the models, while during the test process, the remaining 10% known interactions and all of the non-interacting pairs that were not included in the training set were used to evaluate the prediction performance. All results were summarized over 10 trials of ten-fold cross-validation and expressed as mean $\pm$ SD.

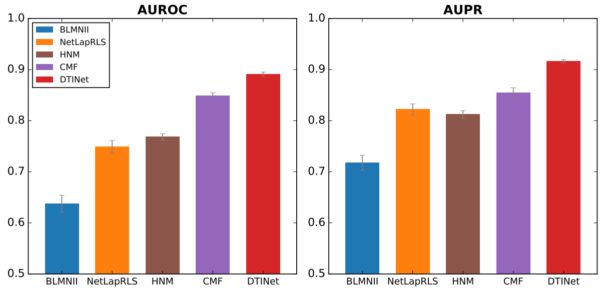

(a)

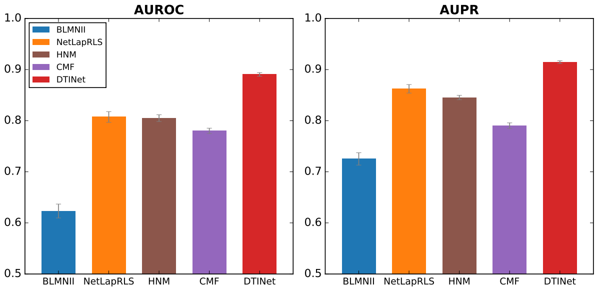

(b)

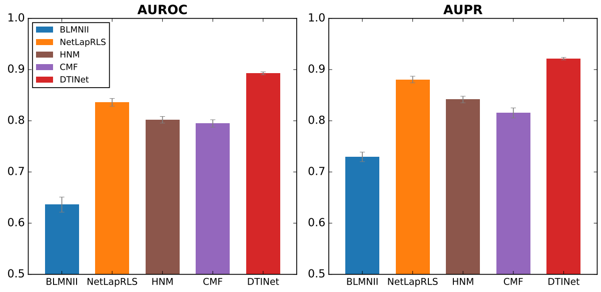

(c)

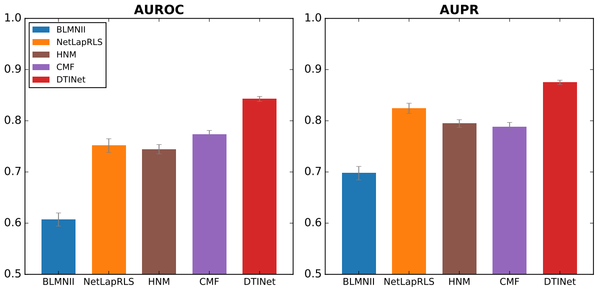

(d)

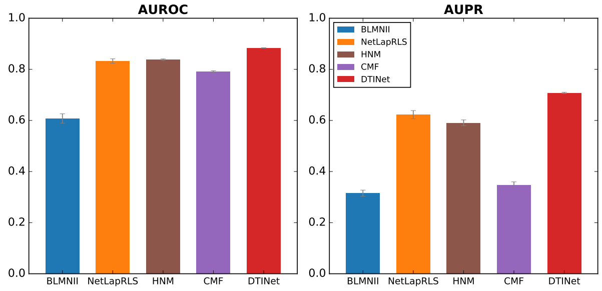

(e)

Supplementary Figure 3: Performance comparisons between different prediction approaches on the datasets after remaining similar drugs or/and targets. (a) The removal of DTIs with similar drugs (Tanimoto coefficients > 60%), (b) The removal of DTIs with the drugs that share similar side-effects (Jaccard similarity scores > 60%). (c) The removal of DTIs with the drugs or proteins associated with similar diseases (Jaccard similarity scores > 60%). (d) The removal of DTIs with either similar drugs (Tanimoto coefficients > 60%) or homologous proteins (sequence identity scores > 40%). These removal operations reduced the number of DTIs from 1,923 to 1,268, 1265, 1077 and 900 in (a), (b), (c) and (d), respectively. In (a)-(d), a matching number of randomly chosen non-interacting drug-target pairs (i.e., negative samples) with the known interacting drug-target pairs (i.e., positive samples) were used as training data. (e) The removal of the DTIs with homologous proteins (sequence similarity scores > 40%) on a skewed dataset, in which known interacting drug-target pairs composed only 10% of the whole test data. All results were summarized over 10 trials of ten-fold cross-validation and expressed as mean ± SD.

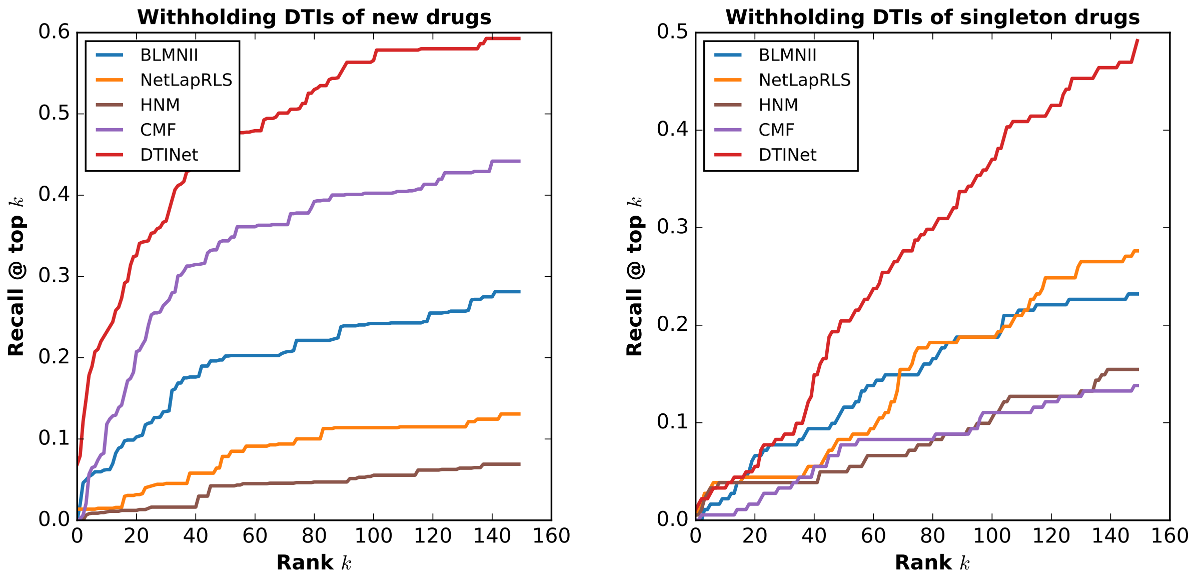

(a) (b)

Supplementary Figure 4: The cumulative distributions of recall @ top-*k* with respect to rank *k* when withholding the DTIs of (a) new drugs or (b) singleton drugs. The new drugs were those with new MOAs (mechanism of actions) discovered within the last five years as of the time that the DrugBank database Version 3.0 (which was used to construct our heterogeneous network) was released. The singleton drugs meant those with only one known interacting target in our dataset. The vertical axis, denoted by recall @ top-*k*, represents the fraction of the true interacting drug-target pairs that were retrieved in the list of top-*k* predictions for a drug. The maximum value of rank *k* was set to 150, which corresponded to roughly 10% of the total number of the targets (1,512).

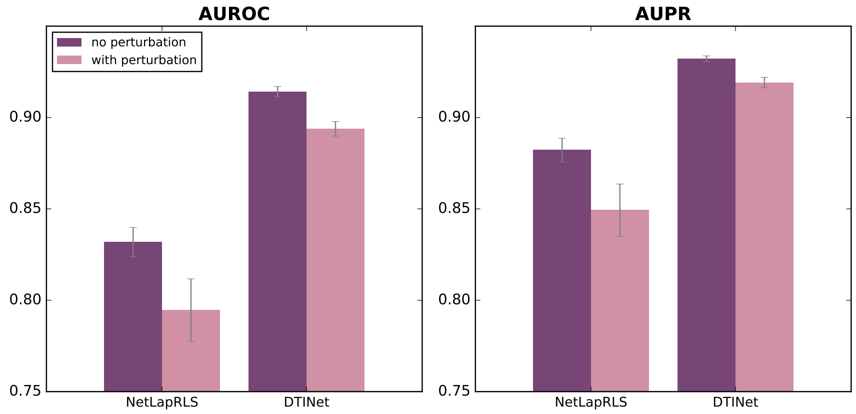

Supplementary Figure 5: Robustness of the prediction performance of DTINet with respect to the random perturbation of edges in the heterogeneous network. A fraction (10%) of randomly selected edges in the heterogeneous network were perturbed, by adding new edges or deleting existing edges. The ground truth drug-target interacting pairs used in the test data were not perturbed. All results were summarized over 10 trials of ten-fold cross-validation and expressed as mean $\pm$ SD.

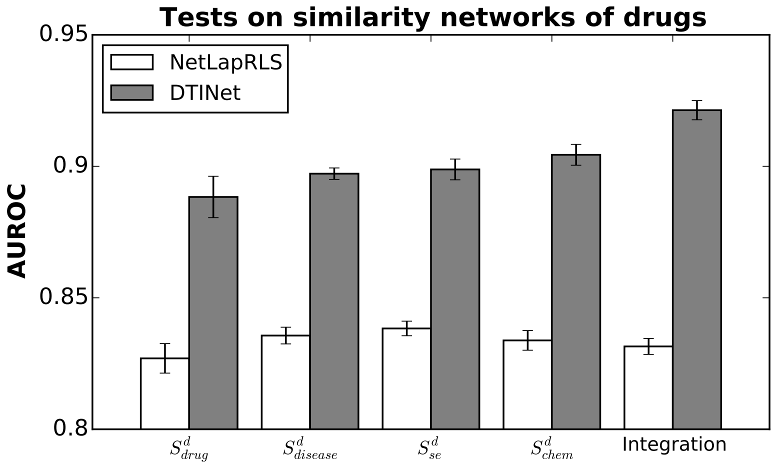

(a)

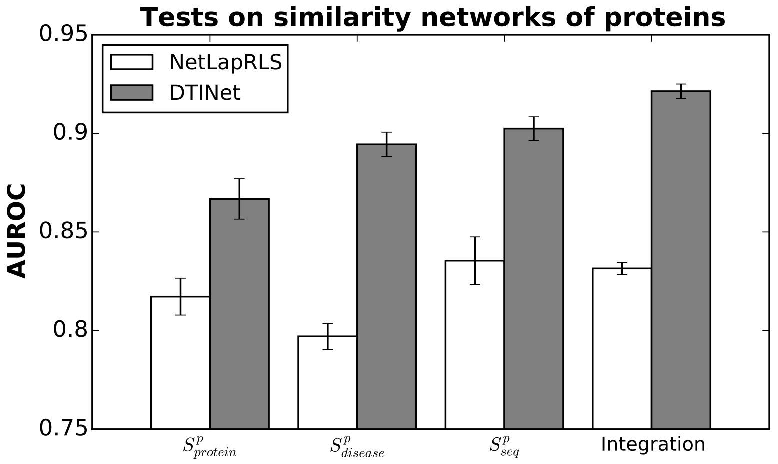

(b)

Supplementary Figure 6: A comparative study on the prediction performance of DTINet and NetLapRLS on individual networks and their integration. (a) The test results on individual similarity networks of drugs and their integration, where S^d_{drug}, $S^d_{disease}$, S^d_{se} and S^d_{chem} represent the similarity networks in which the similarity score between a pair of drug nodes was computed based on the profiles of drug-drug interactions, drug-disease associations, drug-side-effect associations and chemical structures, respectively. (b) Tests on individual similarity networks of proteins and their integration, where $S^p_{protein}$, $S^p_{disease}$ and S^p_{seq} represent the similarity networks in which the similarity score between a pair of protein nodes was computed based on the profiles of protein-protein interactions, protein-disease associations and primary sequences, respectively. An extended version of NetLapRLS (see Supplementary Note 1) was used to combine all similarity networks to perform DTI prediction. All results were summarized over 10 trials of ten-fold cross-validation and expressed as mean ± SD.

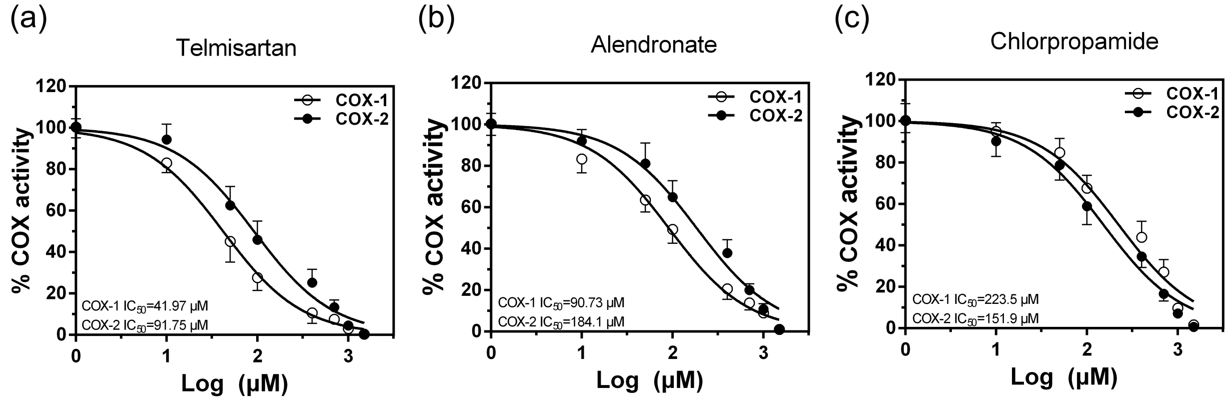

Supplementary Figure 7: The inhibitory effects of telmisartan (a), alendronate (b), and chlorpropamide (c) on COX-1 and COX-2 activities, measured by the human recombinant enzyme assays. The COX-1 (open squares) and COX-2 (closed circles) inhibitions were assessed by measuring the levels of PGE 2.

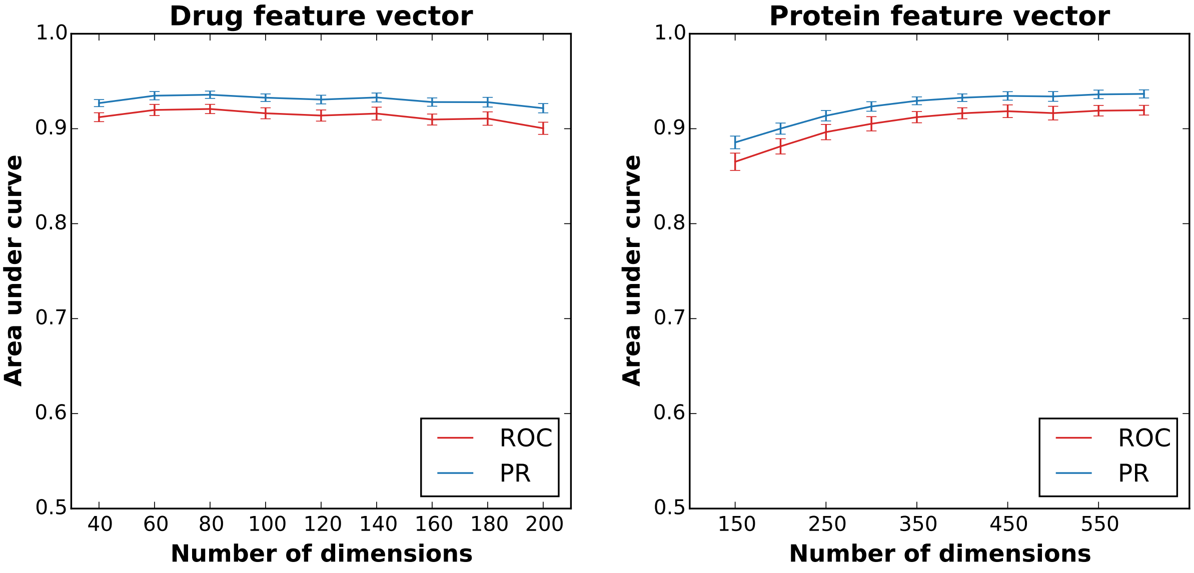

(a) (b)

Supplementary Figure 8: Robustness of DTINet with respect to the number of dimensions of feature vectors. We evaluated the sensitivity of the prediction performance of DTINet with respect to different numbers of dimensions of the feature vectors of drugs (a) and proteins (b). We tested the dimensions of the feature vectors of drugs (f_d) and proteins (f_t) in a range that are roughly equal to 10%-30% of the dimensionality of the original vectors describing the diffusion states. DTINet had stable prediction performance over a wide range the dimensions of the feature vectors. Prediction performance was evaluated in terms of both the area under the receiver operating characteristic curve (ROC) and the area under the precision recall (PR) curve. All results were summarized over 10 trials of ten-fold cross-validation and expressed as mean $\pm$ SD.

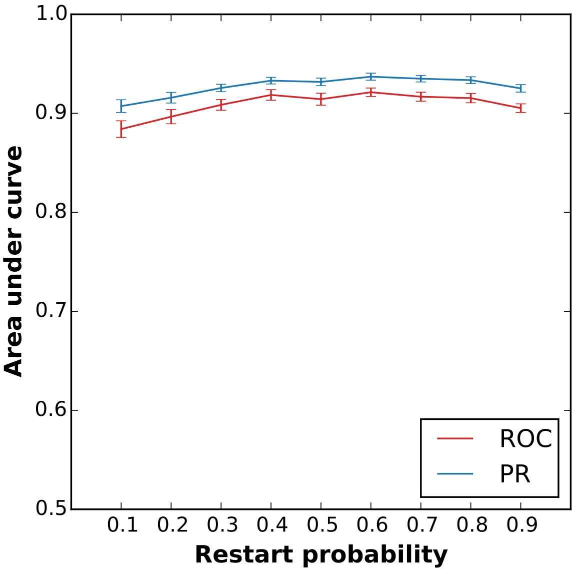

(a)

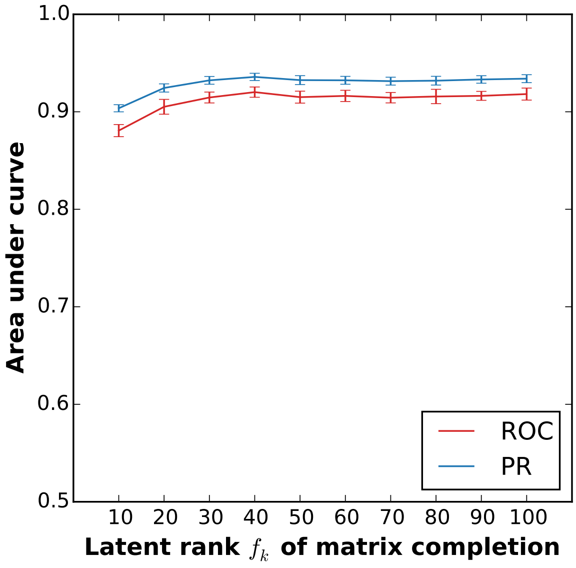

(b)

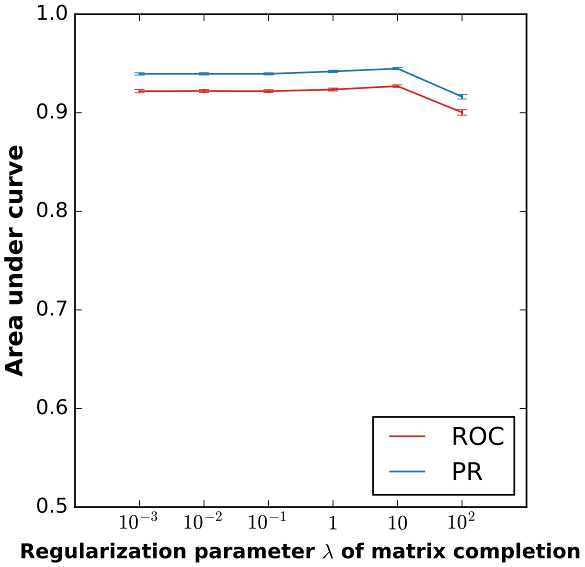

(c)

Supplementary Figure 9: Robustness of the prediction performance of DTINet with respect to different choices of (a) the restart probability, (b) the latent rank of matrix completion, and (c) the regularization parameter. Prediction performance was evaluated in terms of both the area under the receiver operating characteristic curve (ROC) and the area under the precision recall (PR) curve. All results were summarized over 10 trials of ten-fold cross-validation and expressed as mean $\pm$ SD.
